# Transcriptional dynamics during karyogamy in rice zygotes

**DOI:** 10.1242/dev.204497

**Published:** 2025-01-27

**Authors:** Erika Toda, Shizuka Koshimizu, Atsuko Kinoshita, Tetsuya Higashiyama, Takeshi Izawa, Kentaro Yano, Takashi Okamoto

**Affiliations:** ^1^ Department of Biological Sciences, Tokyo Metropolitan University, Hachioji, Tokyo 192-0397, Japan; ^2^ Department of Biological Sciences, The University of Tokyo, Bunkyo, Tokyo 113-0033, Japan; ^3^ Department of Agricultural and Environmental Biology, The University of Tokyo, Bunkyo, Tokyo 113-8657, Japan; ^4^ Department of Life Sciences, Meiji University, Kawasaki, Kanagawa 214-8571, Japan; ^5^ WellGreen-i Co. Ltd., Kawasaki, Kanagawa 215-0007, Japan

**Keywords:** Karyogamy, Rice, Single-cell, Transcriptome, Zygote

## Abstract

Upon fertilization, male and female nuclei fuse to form the zygotic nucleus in angiosperms. Karyogamy is considered to be essential for proper embryogenesis; however, the transcriptional dynamics during karyogamy in plant zygotes remain unclear. In this study, we performed a single-cell transcriptome analysis of rice zygotes at six early developmental stages (15 min, 30 min, 1 h, 2 h, 4 h, and 6 h after gamete fusion) to reveal gene expression profiles during karyogamy in plant zygotes. The time-series RNA-sequencing analysis detected possible *de novo* and altered gene expression in zygotes from 15 min post-fertilization. Fertilization-induced transcription during karyogamy was characterized by protein interaction database and gene ontology (GO) analyses. Furthermore, paternal allele transcription was initiated approximately 30 min to 1 h after gamete fusion, when nuclear fusion begins in the zygote. Some transcripts preferentially expressed in egg cells were downregulated after gamete fusion. Moreover, a dynamic shift from maternal-biased transcripts to bi-parental expression occurred during early zygotic development. These results suggest that transcriptional dynamics during karyogamy plays an initial role in proper and sequential zygotic development and embryogenesis.

## Teaser

**Summary:** Male and female nuclei fuse to form the zygotic nucleus in angiosperms. A time-course single-cell transcriptome analysis reveals gene expression profiles during nuclear fusion in rice zygotes.

## INTRODUCTION

In eukaryotes, the genetic and cytoplasmic contents of male and female gametes combine to form a zygote that transmits genetic materials from the parents to the next generation. In most animals, early embryogenesis depends on maternal mRNAs and proteins stored in the egg, with zygotic genome activation (ZGA) generally beginning after several rounds of cleavage (Tadros and Lipshitz, 2009). Parental genomes do not fuse in mammalian zygotes because two separate spindles are maintained during the first cleavage (Reichmann et al., 2018). By contrast, in angiosperms male and female nuclei fuse to form a zygotic nucleus, implying that male and female genomes become integrated before the first cell division (Mogensen and Holm, 1995; Scholten et al., 2002; Ingouff et al., 2010). There are some controversies regarding parental contributions to early embryogenesis in plants. Several studies have indicated that, similar to most animals, maternal transcripts are major contributors to early embryogenesis in plants, with the maternal-to-zygotic transition occurring several days after fertilization (Vielle-Calzada et al., 2000; Grimanelli et al., 2005; Pillot et al., 2010; Autran et al., 2011). In contrast, the existence of paternal mRNAs and proteins in *Arabidopsis* embryos (Weijers et al., 2001) and maize zygotes (Scholten et al., 2002) indicates that paternal allele transcription occurs during zygotic development and early embryogenesis. Furthermore, parental genomes reportedly contribute equally, as most transcripts are produced from both parental alleles in near-equal quantities during early embryogenesis (Meyer and Scholten, 2007; Nodine and Bartel, 2012). The inhibition of transcription leads to a strong disorder of zygotic elongation and subsequent cell division in *Arabidopsis* and tobacco (Zhao et al., 2011, 2019; Kao and Nodine, 2019), indicating that ZGA is required for proper zygotic development.

Karyogamy, which refers to the fusion of male and female nuclei, results in the formation of a zygotic nucleus in angiosperms. The dynamics of karyogamy have been cytologically examined using plant zygotes isolated from pollinated flowers (Mogensen and Holm, 1995) and produced by an *in vitro* fertilization (IVF) system (Faure et al., 1993; Ohnishi et al., 2014). According to these previous studies, the migration of the sperm nucleus toward the egg nucleus and the integration of male chromatin into the egg nucleus occur during the early stage of zygotes. Analyses of *Arabidopsis* mutants revealed that the nuclear fusion factors BiP and J proteins, which act as molecular chaperones in the endoplasmic reticulum, affect polar nuclei fusion and male and female nuclei fusion (Maruyama et al., 2010, 2014, 2020). Furthermore, two types of nuclear membrane proteins, Sad1-UNC-84 homology (SUN) and gamete-expressed 1 (GEX1), participate in the nuclear fusion process (Hwang et al., 2019; Nishikawa et al., 2020). Aberrant embryo development was observed in *gex1* mutant seeds (Alandete-Saez et al., 2011; Nishikawa et al., 2020). Thus, transcriptional dynamics associated with the fusion of male and female nuclei in zygotes are thought to be crucial for proper zygotic development and embryogenesis in angiosperms.

Molecular studies applying cell type-specific microarrays or RNA sequencing (RNA-seq) technology have revealed comprehensive gene expression profiles and identified transcripts specifically expressed in plant zygotes (Ning et al., 2006; Abiko et al., 2013; Anderson et al., 2017; Chen et al., 2017; Rahman et al., 2019; Zhao et al., 2019). The transcriptomes of gametes, zygotes in early and late developmental stages, and early embryos are consistent with a two-step maternal-to-zygotic transition, and the parental contributions to the zygotic transcriptome are stage dependent in *Arabidopsis* (Zhao et al., 2019). In rice, transcriptome analyses of rice zygotes at specific time points (i.e. 2.5, 5, and 9 h after pollination) indicated that ZGA involves the asymmetrical activation of both parental genomes during zygotic development (Anderson et al., 2017). Furthermore, transcripts showing allele-specific expression in zygotes were identified by a single nucleotide polymorphism (SNP)-based RNA-seq analysis of hybrid zygotes (Anderson et al., 2017; Rahman et al., 2019; Zhao et al., 2019). Notably, *Oryza sativa Apospory Specific Genome Region* (*ASGR*)*–BABY-BOOM LIKE* (*BBML*) *1* (*OsASGR–BBML1*; *PLT6*), a paternally expressed gene in early zygotes, plays crucial roles in the initiation of zygotic development (Khanday et al., 2019; Rahman et al., 2019).

Although karyogamy seems to be essential for the formation of zygotic nucleus and proper zygotic development/embryogenesis in angiosperms, transcriptional dynamics during karyogamy in plant zygotes remain uncharacterized. This is mainly due to the technical challenges associated with isolating zygotes at the earliest post-fertilization stage as well as the difficulty in collecting a sufficient amount of the earliest zygotes for a transcriptome analysis. An IVF system would be suitable for preparation of zygotes to examine the earliest developmental events in zygotes, since it has been reported that a zygote produced by electrofusion of an egg cell with a sperm cell develops into an asymmetric two-celled embryo through proper reorganization of cellular polarity and a globular-like embryo in a manner similar to that *in planta* (Kranz et al., 1995; Sato et al., 2010), and that precise fusion (fertilization) timing of gametes can be set under the microscopes (Kranz and Lörz, 1993; Uchiumi et al., 2007). In this study, we performed a single-cell transcriptome analysis of rice zygotes produced by an IVF system at six early developmental stages (15 min, 30 min, 1 h, 2 h, 4 h, and 6 h after gamete fusion). These analyses revealed gene expression profiles and parental contributions during karyogamy in plant zygotes, shedding light on the upstream molecular mechanisms underlying zygotic development and embryogenesis.

## RESULTS

### Single-cell RNA-seq analysis of rice gametes

**Fig. 1.**
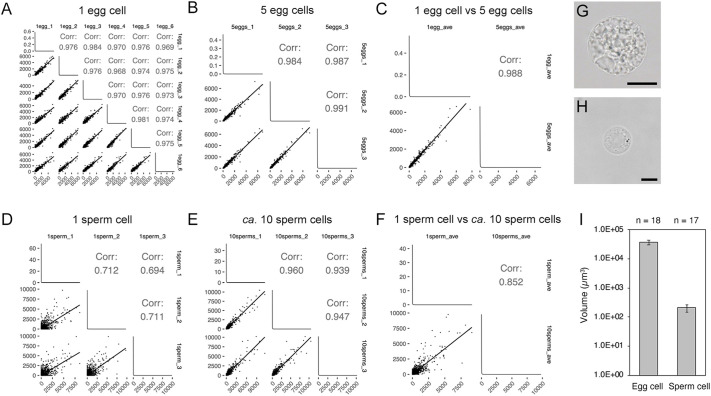
**Reliability of RNA-seq data for single gametes in rice.** (A,B) Scatter plots of TPM values for sample replicates (A: one egg cell, six replicates; B: five egg cells, three replicates). (C) Scatter plot of average TPM values for samples comprising one egg cell and five egg cells. (D,E) Scatter plots of TPM values for sample replicates (D: one sperm cell, three replicates; E: approximately ten sperm cells, three replicates). (F) Scatter plot of average TPM values for samples consisting of one sperm cell and approximately ten sperm cells. (G,H) Example images of an isolated egg cell (G) and an isolated sperm cell (H). (I) Comparison of egg cell and sperm cell volumes. The cell diameter was measured using ImageJ to calculate the cell volume. Data represent mean±s.d. The numbers in the insets (‘Corr:’) represent the Pearson correlation coefficient (*P*<0.001). The correlation coefficient was calculated after manually excluding clear outliers in the data. The numbers of the excluded transcripts as outliers are as follows: (A) 3, (B) 2, (C) 2, (D) 8, (E) 2, and (F) 4. Black lines in plots indicate a linear regression. To assess TPM values in the low-medium abundance range where most of the transcripts are, scatter plots of log values are shown in Fig. S2. Scale bars: 20 µm (G); 5 µm (H).

First, we tested whether single-cell RNA-seq analysis is appropriate for rice gametes. An egg cell and a sperm cell were collected for cDNA synthesis/amplification and library preparation (Fig. S1; Table S1). To assess the reliability of the data for samples consisting of a single gamete, samples comprising multiple gametes were also subjected to RNA-seq analysis (Fig. S1; Tables S1-S3). The transcripts per million (TPM) values were highly correlated among the replicates of the single egg cell sample and between single and five egg cell samples (r>0.95; Fig. 1A-C). In contrast, the correlation between TPM values was lower for the replicates of the single sperm cell sample than for the replicates of the samples consisting of approximately ten sperm cells (Fig. 1D-F). Sperm chromatin was highly condensed, and the sperm cell volume was approximately 1% of that of the egg cell volume (Fig. 1G-I), which may be relevant to the difference in the RNA-seq data quality between a single egg cell and a single sperm cell. On the basis of these results, the RNA-seq data for a single egg cell and approximately ten sperm cells were analyzed further. Because rice egg cells and zygotes are almost similar in size (40-50 µm in diameter; Uchiumi et al., 2007), we obtained RNA-seq data for single zygote samples.

### Transcriptome analysis of rice zygotes during karyogamy

**Fig. 2.**
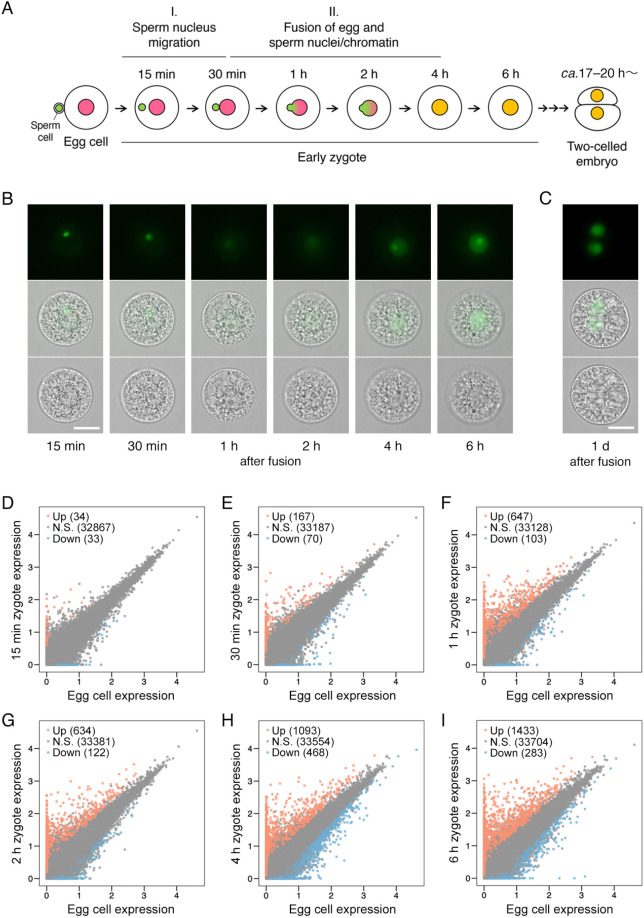
**Identification of transcripts upregulated or downregulated during karyogamy.** (A) Schematic diagram of karyogamic progression during early zygotic development. Pink, green, and orange circles indicate the egg, sperm, and zygotic/embryonic nuclei, respectively. (B) Karyogamic progression in rice zygotes. A sperm cell expressing the H2B-GFP fusion protein was fused with a wild-type egg cell. (C) Two-celled embryo at 1 day after fusion. Top, middle, and bottom panels in B,C present fluorescent, merged fluorescent and brightfield, and brightfield images, respectively. Scale bars: 20 µm. (D-I) Scatter plots of log_10_(TPM+1) values for egg cells and zygotes. Red and blue dots represent upregulated and downregulated DEGs, respectively. N.S., transcripts not selected as DEGs (TPM>0 in both or either egg cells or zygotes). The number of transcripts is presented in parentheses.

Considering karyogamic progression during development of rice zygotes (Fig. 2A-C; Ohnishi et al., 2014), we prepared early zygotes at 15 min, 30 min, 1 h, 2 h, 4 h, and 6 h after gamete fusion. To harvest zygotes at the appropriate karyogamic/developmental stages, zygotes were produced by fusion of a wild-type egg cell with a sperm cell expressing H2B-GFP (Fig. 2B). Single-cell RNA-seq data were generated for zygotes at six developmental stages (Fig. S1; Tables S1, S3, S4). To assess sequencing depth of RNA-seq data for the gametes and zygotes, the number of detectable transcripts (TPM>0) were counted and compared with that in downsampled data (Fig. S3), confirming that the sequencing depth in this study is in the appropriate range for further analysis. Principal component analysis (PCA) showed that the RNA-seq data of egg cells and sperm cells were clearly separated, with the zygote samples gradually shifting along developmental stages (Fig. S4). The TPM values were relatively highly correlated among sample replicates in early zygotes (Figs S5, S6). The partially low correlation among the sample replicates of the zygotes at 4 and 6 h after gamete fusion may be due to individual differences in their developmental stages. Furthermore, to confirm whether the RNA-seq datasets are reliable, the expression profiles of representative genes involved in fertilization and subsequent development were verified on the basis of TPM values and reverse transcription-quantitative PCR (RT-qPCR) data, which enabled the monitoring of gamete-specific and fertilization-induced expression (Fig. S7). Next, differentially expressed genes (DEGs) between the egg cell and the zygote at each stage were assessed and screened to identify transcripts with upregulated or downregulated expression after fertilization (Fig. 2D-I; Tables S5, S6; Fig. S8). DEGs were identified in zygotes even at 15 and 30 min after gamete fusion. Additionally, the number of DEGs gradually increased during early zygotic development, reflecting the shift in gene expression profiles from the egg cell to the zygote soon after fertilization. Moreover, characteristic GO terms were assigned to the upregulated DEGs, although there was some overlap in the major GO terms among the early zygote developmental stages (Fig. S9).

### *De novo* gene expression is initiated in rice zygotes soon after fertilization

**Fig. 3.**
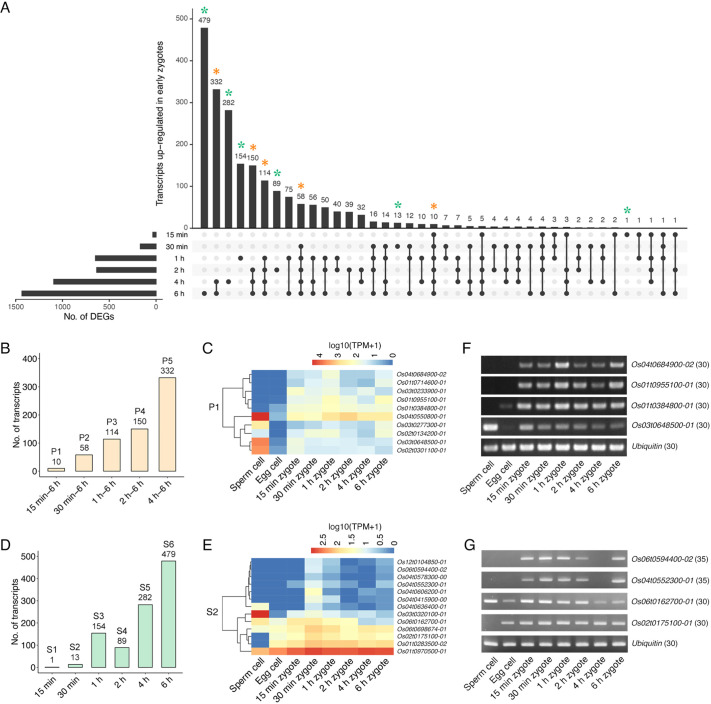
**Initiation of *de novo* gene expression in the earliest zygote stage.** (A) Set visualization of upregulated DEGs in each zygote stage. Orange and green asterisks indicate persistent and stage-specific upregulation, respectively. (B) Transcripts exhibiting persistent upregulation extracted from the data shown in A. These categories were designated as P1-P5. (C) Heat maps for TPM values of P1 transcripts in sperm cells, egg cells, and early zygotes. Upper and lower clades indicate possible *de novo* expression and sperm cell-enriched transcription, respectively. (D) Transcripts exhibiting stage-specific upregulation extracted from the data shown in A. These categories were designated as S1-S6. (E) Heat maps for TPM values of S2 transcripts in sperm cells, egg cells, and early zygotes. (F,G) Expression patterns of representative P1 (F) and S2 (G) transcripts confirmed by semi-quantitative RT-PCR. Ubiquitin was used as an internal control. Numbers in parentheses indicate the number of PCR cycles.

To analyze the upregulated DEGs further, persistent and stage-specific DEGs among six developmental stages were identified (Fig. 3A; Table S7). As indicated by orange asterisks in Fig. 3A, we divided the persistent DEGs that were continuously upregulated (relative to their expression in egg cells) into the following five categories: 15 min-6 h, 30 min-6 h, 1-6 h, 2-6 h, and 4-6 h (Fig. 3B; P1-P5). The number of persistent upregulated DEGs increased as zygotic development progressed. The TPM value of each transcript in egg cells, sperm cells, and early zygotes at six developmental stages showed that P1 contained both sperm cell-enriched transcripts and transcripts reflecting possible *de novo* expression from 15 min after gamete fusion (Fig. 3C). Similarly, transcripts associated with possible *de novo* gene expression were detected in P2-P5 (Fig. S10A-D). These results indicate that *de novo* gene expression was initiated in rice zygotes soon after fertilization. Furthermore, the stage-specific upregulated DEGs were divided into six categories, indicated by green asterisks in Fig. 3A and designated as S1-S6 in Fig. 3D. The S2-S6 transcripts were transiently upregulated (Fig. 3E; Fig. S10E-H), indicative of altered gene expression during karyogamy in early zygotes. The expression profiles of representative P1 and S2 transcripts were confirmed by semi-quantitative RT-PCR (Fig. 3F,G).

### Characterization of fertilization-induced transcription during karyogamy

**Fig. 4.**
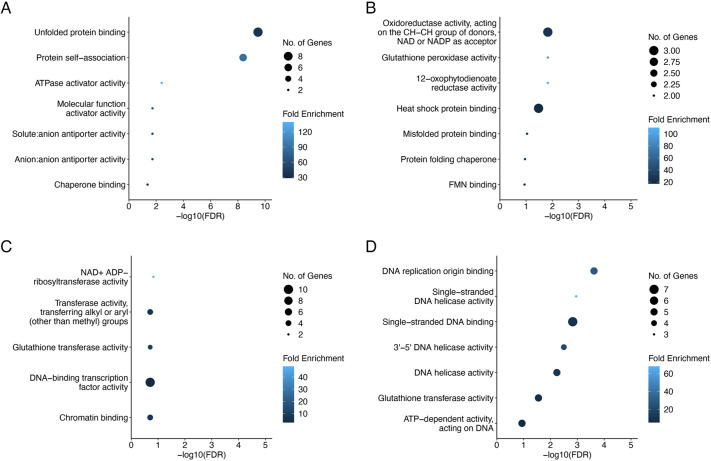
**Characteristic GO terms among the upregulated transcripts in early zygotes.** (A-D) Characteristic GO terms (molecular function) in transcript categories P2 (A; 58 transcripts persistently upregulated in 30 min-6 h), P3 (B; 114 transcripts persistently upregulated in 1 h-6 h), P4 (C; 150 transcripts persistently upregulated in 2 h-6 h), and P5 (D; 332 transcripts persistently upregulated in 4 h-6 h). Top GO terms are represented for each transcript category.

Because the persistent upregulated DEGs (Fig. 3B) are assumed to be involved in upstream molecular mechanisms influencing zygotic development, a gene network analysis was conducted to examine putative interactions among the proteins encoded by P1-P5 transcripts (Fig. S11). The interacting proteins in this network were associated with the following four Kyoto Encyclopedia of Genes and Genomes (KEGG) pathways: ‘Protein processing in endoplasmic reticulum’, ‘Glutathione metabolism’, ‘Cysteine and methionine metabolism’, and ‘DNA replication’. Furthermore, GO analysis of the P2-P5 transcripts was performed to identify sequential GO terms. Transcripts related to ‘Unfolded protein binding’ and ‘Protein self-association’ were highly enriched in P2 (Fig. 4A). The transcripts in P3 included those associated with ‘glutathione peroxidase activity’ (Fig. 4B), which is consistent with the reported *de novo* expression of genes encoding glutathione peroxidases responsible for decreasing reactive oxygen species levels in rice zygotes (Rattanawong et al., 2021). In addition, GO terms related to chromatin/DNA binding or DNA helicase activity were preferentially enriched in P4 and P5 (Fig. 4C,D), which was in accordance with previous studies that indicated that upregulated genes in rice zygotes are involved in chromatin/DNA organization and assembly (Abiko et al., 2013) and genes encoding transcriptional regulators from various families are activated in maize zygotes (Chen et al., 2017). Considered together, these results mainly indicate that genes related to metabolism/protein interaction and to DNA/chromatin organization are induced soon after gamete fusion and at the late karyogamic stage in early zygotes, respectively, suggesting that genes/transcripts with different functional categories are upregulated in a sequential and stepwise manner.

### Transcripts preferentially expressed in egg cells are downregulated after fertilization

**Fig. 5.**
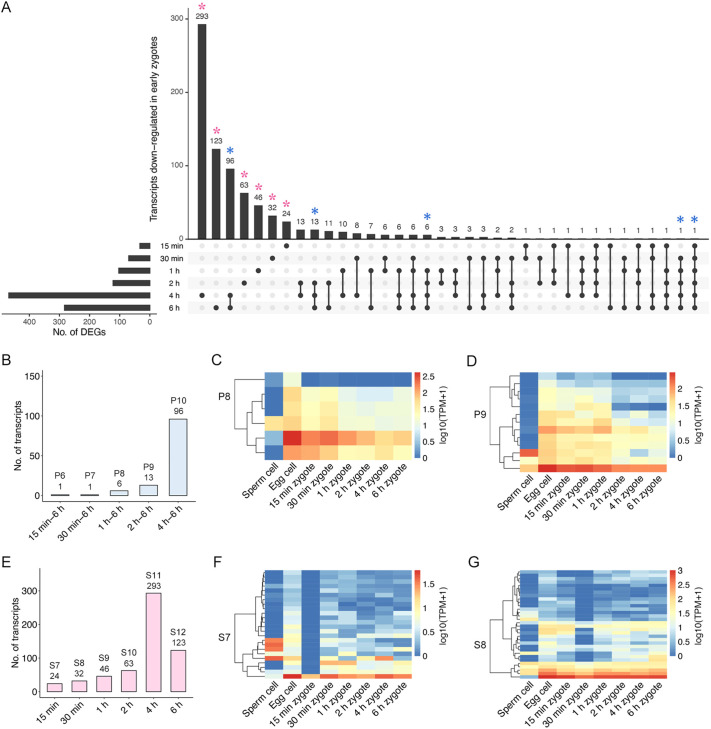
**Expression levels of transcripts specifically expressed in egg cells are downregulated after fertilization.** (A) Set visualization of downregulated DEGs in each zygote stage. Blue and pink asterisks indicate persistent and stage-specific downregulation, respectively. (B) Transcripts exhibiting persistent downregulation extracted from the data shown in A. These categories were designated as P6-P10. (C,D) Heat maps for TPM values of P8 and P9 transcripts in sperm cells, egg cells, and early zygotes. (E) Transcripts exhibiting stage-specific downregulation extracted from the data shown in A. These categories were designated as S7-S12. (F,G) Heat maps for TPM values of S7 and S8 transcripts in sperm cells, egg cells, and early zygotes.

For further analysis of the downregulated DEGs, persistent and stage-specific DEGs among six developmental stages were identified (Fig. 5A; Table S8). As indicated by blue asterisks in Fig. 5A, the persistent DEGs that were continuously downregulated (relative to their expression in egg cells) were divided into the following five categories: 15 min-6 h, 30 min-6 h, 1-6 h, 2-6 h, and 4-6 h (Fig. 5B; P6-P10). The number of persistent downregulated DEGs increased as zygotic development progressed. On the basis of the TPM value of each transcript in egg cells, sperm cells, and early zygotes at six developmental stages, the expression of P8-P10 transcripts preferentially expressed in egg cells gradually decreased during early zygotic development (Fig. 5C,D; Fig. S12A). In addition, stage-specific downregulated DEGs were divided into six categories, indicated by pink asterisks in Fig. 5A and designated as S7-S12 in Fig. 5E. The S7-S11 transcripts showed transient downregulation (Fig. 5F,G; Fig. S12B-E), supporting the suppression of gene expression during karyogamy in early zygotes.

### Changes of the maternal:paternal transcript ratio during karyogamy

**Fig. 6.**
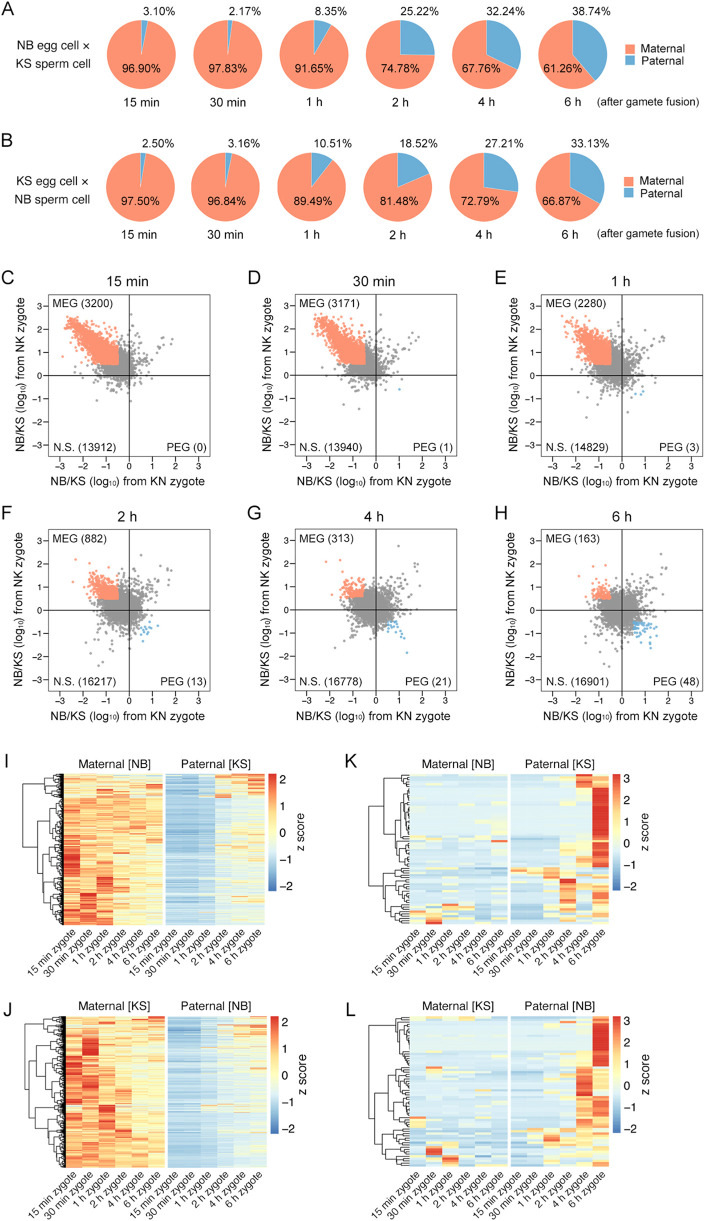
**Allelic transcriptional dynamics during karyogamy in early zygotes.** (A) Ratio of parental RNA-seq reads (maternal:paternal) in zygotes produced by the fusion of an NB egg cell and a KS sperm cell. (B) Ratio of parental RNA-seq reads (maternal:paternal) in zygotes produced by the fusion of a KS egg cell and an NB sperm cell. (C-H) Scatter plots for TPM ratios (NB/KS) of transcripts with SNP-containing reads. Red and blue dots indicate MEGs and PEGs, respectively. The number of MEGs, PEGs, and N.S. (comparable transcripts not selected as MEGs or PEGs) in each zygote stage is provided in parentheses. (I,J) Heat maps for row-scaled allelic TPM values of the 3632 MEGs detected as shown in C-H in NK zygotes (I) and KN zygotes (J) at 15 min-6 h after gamete fusion. (K,L) Heat maps for row-scaled allelic TPM values of the 66 PEGs detected as shown in C-H in NK zygotes (K) and KN zygotes (L) at 15 min-6 h after gamete fusion.

To investigate allelic expression patterns during karyogamy in early zygotes, a SNP-based RNA-seq analysis was conducted using intersubspecific rice zygotes at 15 min, 30 min, 1 h, 2 h, 4 h, and 6 h after gamete fusion (Figs S13-S15; Tables S1, S3, S9). These zygotes were produced by reciprocal fusion between a Nipponbare (NB) egg cell and a Kasalath (KS) sperm cell (NB×KS, which is hereafter abbreviated as NK) and between a KS egg cell and an NB sperm cell (KS×NB, which is hereafter abbreviated as KN). The RNA-seq data were simultaneously mapped onto NB and KS transcript sequences, and then the read counts were compared between NB and KS using 17,112 comparable transcripts (Table S10). In the NK zygotes at 15 and 30 min after gamete fusion, 96.9-97.8% of the reads were derived from maternal alleles. The remaining paternal reads (2.2-3.1%) were likely derived from a sperm cell (Fig. 6A). The proportion of paternal reads increased from 1 h after gamete fusion. Accordingly, paternal allele transcription was activated at approximately 30 min to 1 h after gamete fusion; this timing coincided with the initiation of the fusion of male and female nuclei in zygotes (Fig. 2A,B). In addition to the activation of transcription from paternal alleles, transcriptional suppression of maternal alleles and degradation of maternal transcripts may also affect the reduced proportion of maternal reads. Similar results were obtained for KN zygotes (Fig. 6B), implying that the change in the maternal:paternal transcript ratio was due to differences in parental alleles, and was independent of the cultivar.

### Genome-wide transition from maternal-biased transcripts to bi-parental allele expression

On the basis of the TPM ratio, maternally biased transcripts, which are supposed to be primarily derived from an egg cell, were detected in the zygotes at 15 and 30 min after gamete fusion (Fig. 6C,D). Thereafter, genome-wide expression profiles gradually shifted from maternal-biased transcripts to bi-allelic expression during the progression of early zygotic development (Fig. 6E-H). The genome-wide transition resulted in a substantial decrease in the number of maternally expressed genes (MEGs) and a slight increase in the number of paternally expressed genes (PEGs) along the progression of zygotic development (Fig. 6C-H). Allelic transcriptional dynamics of 3632 MEGs and 66 PEGs detected in Fig. 6C-H without overlapping were monitored in early stage of zygotes. As a result, a decrease in expression level from maternal alleles and an initiation of transcription from paternal alleles were shown in the MEGs (Fig. 6I,J), with gene expression in a paternal-allele dependent manner in the PEGs (Fig. 6K,L). These results indicate that transcriptional suppression of maternal allele and/or degradation of maternal transcripts occur during early zygotic development with gradual activation of paternal alleles. To analyze the MEGs further, persistent MEGs among six developmental stages were identified and designated as M1-M5 (Fig. S16A). For most of the MEGs, we detected a transition to bi-allelic expression in the zygotes at approximately 30 min to 2 h after gamete fusion. The KEGG analysis of the MEG categories specifically detected ‘Metabolic pathways’ or ‘Ribosome’ in M2-M5 (Fig. S16B-F). Furthermore, the allelic expression patterns of M1-M5 in early zygotes commonly showed that transcription from paternal alleles was initiated from 1 h after gamete fusion (Fig. S17), which was consistent with the change in the parental transcript ratio (Fig. 6A,B).

## DISCUSSION

In this study, we obtained single-cell RNA-seq datasets of rice zygotes at early developmental stages (i.e. 15 min, 30 min, 1 h, 2 h, 4 h, and 6 h after gamete fusion). The data revealed the following transcriptional dynamics during karyogamy: (1) *de novo* and altered gene expression was possibly initiated in zygotes shortly after fertilization; (2) genes/transcripts encoding proteins with diverse molecular functions were upregulated in a sequential and stepwise manner; (3) some of the transcripts preferentially expressed in egg cells were gradually downregulated after fertilization; (4) paternal allele transcription was initiated approximately 30 min to 1 h after gamete fusion, when nuclear fusion begins in the zygote; (5) a global shift of transcriptomics occurs from maternal-biased transcripts to bi-parental expression. In addition, the RNA-seq datasets enabled us to explore cell type-specific expression in gametes/zygotes and altered gene expression during karyogamy in early zygotes (Figs 3, 5; Figs S7, S10, S12). Notably, the relative expression level of *OsGEX1*, an ortholog of *AtGEX1*, which plays role in karyogamy in *Arabidopsis* (Nishikawa et al., 2020), transiently increased during the progression of karyogamy (Fig. S7). Furthermore, the allelic transcriptional analysis clarified the timing of the onset of paternal allele transcription during karyogamy (Fig. 6; Fig. S17). Our transcriptomic results support the findings of cytological studies showing that paternal mRNA synthesis coincides with male chromatin decondensation in maize zygotes (Scholten et al., 2002).

To confirm that the upregulation of genes in zygotes is caused by fusion of an egg cell with a sperm cell, not by artificial treatment of cells such as electrofusion and cell culture, isolated egg cells were subjected to mock fusion (electrofusion without a sperm cell) and subsequent cell culture, and then the expression profiles of the genes that have been reported to be induced in rice zygotes via fertilization (Abiko et al., 2013) were investigated (Fig. S18A). The results clearly showed that fertilization-induced genes are not expressed in mock-treated egg cells (Fig. S18B), indicating that the changes of gene expression profiles in early zygotes are induced by gamete fusion and karyogamy, not by stimuli from electrofusion and subsequent cell culture. To assess the methods for searching DEGs, we also performed DEG analysis between egg cells and zygotes using different approaches: samr (Li and Tibshirani, 2013) in addition to TCC (Sun et al., 2013) (Fig. S18A,C). Thus, application of multiple statistical analyses would be effective to detect genes/transcripts showing apparent significance toward further analysis.

An egg cell establishes a static state that is maintained until fertilization. Upon fertilization, egg cells shift to an active state with dynamic changes in metabolic and developmental activities. Entry of a sperm cell into the egg cell triggers an increase in cytosolic Ca^2+^ (Denninger et al., 2014; Hamamura et al., 2014), serving as a key signaling molecule that activates egg cells (reviewed by Chen et al., 2015). In this study, characteristic GO terms such as ‘Calcium ion binding’ and ‘Calmodulin binding’ were enriched in the upregulated DEGs (Fig. S9). Additionally, transcripts encoding calmodulin-like protein (*OsCML31*) and putative hypersensitive reaction associated Ca^2+^-binding protein (*Os03g0310800*) were detected in P1 and P2, respectively, and identified as MEGs (Fig. 3; Table S7). P1 and P2 transcripts were persistently upregulated before or upon nuclear fusion in early zygotes, suggesting that such transcripts rapidly upregulated shortly after fertilization are involved in fertilization-induced egg cell activation.

SNP-based transcriptome analyses for intersubspecific rice zygotes indicated that maternal reads occupied approximately 97% of total reads in zygotes at 15 and 30 min after gamete fusion, and that the percentage of maternal reads decreased in approximately 90% and 70% in zygotes at 1 h and 4 h after gamete fusion, respectively (Fig. 6A,B). This suggests that paternal allele transcription can be activated at approximately 30 min to 1 h after gamete fusion. As for maternal:paternal transcript ratio in rice zygotes, Anderson et al. (2017) reported that approximately 95-99% of the reads were derived from maternal alleles in zygotes isolated from flowers at 2.5 h post-pollination between cv. Kitaake and cv. IR50. Estimated from the present results, zygotes located in the embryo sac of ovaries at 2.5 h post-pollination will correspond to zygotes at 15 min to 1 h after gamete fusion based on the duration from pollination to fertilization. Alternatively, putative low pollination efficiency may be a reason for partial contamination of egg cells during the isolation procedures of pollinated egg cells (zygotes), resulting in the increase of maternal reads. In addition to transcriptomics during karyogamy in early zygotes, further investigations of transcriptional dynamics during middle and late developmental stages in zygotes and comparison with other studies will provide comprehensive insights into the gene expression regulation underlying zygotic development and embryogenesis.

Because of the integration of the male and female genomes by karyogamy, the transition in parental contributions in zygotes can be partially explained by differences and changes in the epigenetic states of parental genomes. In rice, global non-CG methylation levels differ considerably between male and female gametes. In contrast, the non-CG methylation levels are balanced between both parental genomes in the embryo, suggestive of the reprogramming of DNA methylation after fertilization (Kim et al., 2019). In addition, allele-specific DNA methylation in rice hybrid zygotes implies that paternal DNA methylation is remodeled to match the maternal pattern during zygotic genome reprogramming (Liu et al., 2023). As for the role of histone modifications, H3K27me3 is replaced by H3K4me3 in *Arabidopsis* sperm cells, likely leading to the reprogramming of the paternal epigenome toward early embryonic development (Borg et al., 2020). Furthermore, chromatin conformation capture (3C) and high-throughput 3C (Hi-C) assays have revealed the three-dimensional (3D) genome structures in the rice egg cells, sperm cells, and unicellular zygotes (Zhou et al., 2019). Comparative analysis of 3D genome structures has detected a compact silent center in egg cells and unicellular zygotes, but not in sperm cells. Compact silent center reorganization following fertilization may be involved in the regulation of ZGA (Zhou et al., 2019). These studies suggest that transcriptional dynamics during karyogamy can be partially explained by epigenetic reprogramming, although changes in the epigenetic states of both parental genomes during karyogamy in zygotes remain unclear. Further investigation is required to elucidate the relationship between the epigenetic/chromatin state and gene expression regulation during early zygotic development. The findings of such research will further clarify the molecular basis of zygotic development and embryogenesis in plants.

## MATERIALS AND METHODS

### Plant materials

*Oryza sativa* L. cv. Nipponbare (NB) and cv. Kasalath (KS) were grown in an environmental chamber (K30-7248; Koito Industries) at 26°C with a 13-h light/11-h dark photoperiod. Transformed rice plants (cv. NB) expressing the histone H2B-GFP fusion protein were prepared as previously described (Abiko et al., 2013). *Agrobacterium tumefaciens* LBA4404 transformed with the *Ubi* promoter::*H2B-GFP* construct (Abiko et al., 2013) was used for the transformation of rice plants (cv. KS), which involved the co-cultivation of scutellum tissue with *A. tumefaciens* as described by Toki et al. (2006).

### Isolation and electrofusion of gametes

Egg cells and sperm cells were isolated from rice flowers as described previously (Uchiumi et al., 2006). Zygotes were produced by electrofusion of an egg cell with a sperm cell according to a published procedure (Uchiumi et al., 2007). Intersubspecific zygotes were produced through the electrofusion of gametes isolated from NB and KS flowers. Details regarding the isolation and electrofusion of gametes have been described by Toda et al. (2016).

### Microscopy

Gametes and zygotes were examined using an IX-71 inverted fluorescence microscope (Olympus), with excitation and emission wavelengths of 460-490 and 510-550 nm, respectively (U-MWIBA2 mirror unit; Olympus). Sperm cells were observed under a STELLARIS 8 confocal microscope (Leica Microsystems). Karyogamic progression in zygotes and development of two-celled embryos were observed using a BZ-X800 inverted fluorescence microscope (Keyence), with excitation and emission wavelengths of 450-490 and 500-550 nm, respectively (BZ-X Filter GFP; Keyence).

### Sampling of gametes and zygotes for RNA-seq analysis

Isolated egg cells and sperm cells were transferred to droplets of mannitol solution adjusted to 370 mOsmol kg^−1^ H_2_O on coverslips. Gametes were washed three or four times by transferring the cells to fresh droplets of mannitol solution, after which they were transferred to the lysis buffer supplied in a SMART-Seq HT Kit (Takara Bio). The lysates were used to synthesize cDNA or immediately frozen in liquid nitrogen and stored at −80°C until used.

Zygotes were prepared by electrofusion of isolated gametes (Uchiumi et al., 2007). Sperm cells expressing the histone H2B-GFP fusion protein were used for gamete fusion to monitor karyogamic progression in zygotes. The zygotes were incubated in droplets of mannitol solution adjusted to 370 mOsmol kg^−1^ H_2_O on coverslips until sampling. At the indicated time (15 min, 30 min, 1 h, 2 h, 4 h, and 6 h after gamete fusion), fluorescent signal from H2B-GFP in zygotes was observed, and the zygotes showing typical karyogamy progression (Fig. 2A-C; Ohnishi et al., 2014) were transferred to the lysis buffer supplied in the SMART-Seq HT Kit after being washed as described above. The lysates were immediately frozen in liquid nitrogen and stored at −80°C until used.

### cDNA synthesis, library preparation, and RNA-seq analysis

cDNA and library preparation were performed as previously reported (Deushi et al., 2021). Briefly, cDNA was synthesized and amplified from cell lysates using the SMART-Seq HT Kit. The amplified cDNA was purified using an Agencourt AMPure XP (Beckman Coulter). The quality and quantity of the purified cDNA were determined using a Qubit 3 Fluorometer with a Qubit dsDNA HS Assay Kit (Thermo Fisher Scientific) and an Agilent 2100 Bioanalyzer with a High Sensitivity DNA chip (Agilent Technologies). Libraries were prepared from the purified cDNA using a Nextera XT DNA Library Prep Kit (Illumina), after which they were purified using an Agencourt AMPure XP. The quality and quantity of the library were determined as described above. Libraries were sequenced using an Illumina HiSeq platform to produce 150 bp paired-end reads.

### Transcriptome data analysis for gametes and isogenic zygotes

Quality of the Illumina reads was evaluated using FastQC (v.0.11.8; https://www.bioinformatics.babraham.ac.uk/projects/fastqc/). The reads were pre-processed using Cutadapt (v.2.10; Martin, 2011) to remove adapters, poly-A sequences, and low-quality sequences. The pre-processed reads were mapped to the NB transcript sequences in RAP-DB (Sakai et al., 2013; Kawahara et al., 2013) and read counts and TPM values were calculated using RSEM (v.1.3.1; Li and Dewey, 2011) with Bowtie2 (v.2.3.5.1; Langmead and Salzberg, 2012). Read counts were compared between egg cells and zygotes, and transcripts with a false discovery rate (FDR)<0.05 were extracted as DEGs using TCC (Sun et al., 2013) in R software. Intersecting sets of DEGs were visualized using UpsetR (Conway et al., 2017) in R software. Multiple protein interactions were analyzed using STRING (v.12.0; Szklarczyk et al., 2023). GO analysis was performed using ShinyGO (v.0.66; Ge et al., 2020). Expression profiles of each transcript category were visualized using pheatmap (https://cran.r-project.org/package=pheatmap). To assess the suitability of the methods for searching DEGs, read counts of egg cells and zygotes were compared to extract transcripts with FDR<0.05 using samr (v.3.0; nperm=2000; Li and Tibshirani, 2013).

To assess sequencing depth of the transcriptome data, the pre-processed reads were downsampled using Seqkit (v.2.8.2; Shen et al., 2016). The pre-processed and downsampled reads were mapped to the NB transcript sequences and read counts and TPM values were calculated as described above. Read counts were compared between egg cells and zygotes at 6 h after gamete fusion, and transcripts with FDR<0.05 were extracted as DEGs using TCC (v.1.44.0; Sun et al., 2013) in R software.

### Sampling of intersubspecific zygotes for RNA-seq analysis

Intersubspecific zygotes were prepared by electrofusion of an NB egg cell and a KS sperm cell (NB×KS; NK zygote) or a KS egg cell and an NB sperm cell (KS×NB; KN zygote). NB and KS sperm cells expressing the histone H2B-GFP fusion protein were used for gamete fusion to monitor karyogamic progression in zygotes. Sampling procedures were the same as those used for the isogenic zygotes described above. cDNA was synthesized, libraries were constructed, and an RNA-seq analysis was completed as described above.

### Transcriptome data analysis for intersubspecific zygotes

To estimate the parental origin of transcripts, reads were mapped to the transcript sequences from NB (Sakai et al., 2013; Kawahara et al., 2013) and KS (Sakai et al., 2014) using Bowtie2. The reads were classified on the basis of the number of mismatches with the reference sequences using HomeoRoq (Akama et al., 2014). Read counts were calculated using featureCounts (v.2.0.0; Liao et al., 2014), and the data for comparable transcripts between NB and KS were used to calculate the maternal:paternal transcript ratio. The TPM value was calculated using read counts according to a known formula (Li et al., 2010; Wagner et al., 2012). Maternally and paternally expressed transcripts were determined as follows: NB/KS [log_10_(TPM)]>0.5 or <−0.5, referring to a previous report (Zhao et al., 2019). Intersecting sets of MEGs were visualized using UpsetR (Conway et al., 2017) in R software. KEGG analysis was performed using ShinyGO (v.0.76; Ge et al., 2020). Expression profiles of each transcript category were visualized using pheatmap (https://cran.r-project.org/package=pheatmap).

### Semi-quantitative RT-PCR

For both gametes and zygotes, cDNA was synthesized as described above. For the PCR analysis presented in Fig. 3F, 1 µl cDNA (200 pg/µl) was used as the template in a 50 µl reaction volume containing 0.3 µM primers and KOD-FX DNA polymerase (Toyobo). The PCR conditions were as follows: 30 or 35 cycles of 98°C for 10 s, 55°C for 30 s, and 68°C for 1 min. The PCR products were subjected to electrophoresis on 2% agarose gels and visualized with ethidium bromide staining. For the PCR analysis presented in Fig. 3G and Fig. S18B, 1 µl cDNA (200 pg/µl) was used as the template in a 20 µl reaction volume containing 0.3 µM primers and KOD-FX DNA polymerase (Toyobo). The PCR conditions were as follows: 30 or 35 cycles of 98°C for 10 s, 60°C for 30 s, and 68°C for 30 s. The PCR products were subjected to electrophoresis on 2% agarose gels and visualized with Midori Green Advance (Nippon Genetics). A ubiquitin gene (*Os02g0161900*) was selected as an internal control. Primer information is presented in Table S11.

### RT-qPCR

cDNA of gametes and zygotes was synthesized as described above and used as the template for the qPCR analysis. Briefly, 1 µl cDNA (20 pg/µl) was used as the template in a 20 µl reaction volume containing 6 pmol primers and THUNDERBIRD Next SYBR qPCR Mix (Toyobo). The PCR conditions were as follows: 45 cycles of 95°C for 5 s and 60°C for 30 s. A ubiquitin gene (*Os02g0161900*) served as an internal control. Fold changes in the relative abundance of transcripts were calculated according to the 2^−ΔΔCt^ method (Livak and Schmittgen, 2001). All RT-qPCR analyses were performed using three biological replicates, each comprising three technical replicates. Primer information is presented in Table S11.

## Supplementary Material



10.1242/develop.204497_sup1 Supplementary information

Table S5. Expression profiles of upregulated DEGs during karyogamy in rice zygotes.

Table S6. Expression profiles of downregulated DEGs during karyogamy in rice zygotes.

Table S7. Expression profiles of transcripts upregulated with persistent or stage-specific manner.

Table S8. Expression profiles of transcripts downregulated with persistent or stage-specific manner.
