# Correction: Transcriptional dynamics during karyogamy in rice zygotes

**DOI:** 10.1242/dev.204691

**Published:** 2025-02-11

**Authors:** Erika Toda, Shizuka Koshimizu, Atsuko Kinoshita, Tetsuya Higashiyama, Takeshi Izawa, Kentaro Yano, Takashi Okamoto

There was an error in *Development* (2025) **152**, dev204497 (doi:10.1242/dev.204497).

The legend for Fig. 2 was incorrect. The corrected and original legends are shown below.

Corrected:

**Fig. 2. Identification of transcripts upregulated or downregulated during karyogamy.** (A) Schematic diagram of karyogamic progression during early zygotic development. Pink, green, and orange circles indicate the egg, sperm, and zygotic/embryonic nuclei, respectively. (B) Karyogamic progression in rice zygotes. A sperm cell expressing the H2B-GFP fusion protein was fused with a wild-type egg cell. (C) Two-celled embryo at 1 day after fusion. Top, middle, and bottom panels in B,C present fluorescent, merged fluorescent and brightfield, and brightfield images, respectively. Scale bars: 20 μm. (D-I) Scatter plots of log10(TPM+1) values for egg cells and zygotes. Red and blue dots represent upregulated and downregulated DEGs, respectively. N.S., transcripts not selected as DEGs (TPM>0 in both or either egg cells or zygotes). The number of transcripts is presented in parentheses.

Original:

**Fig. 2. Identification of transcripts upregulated or downregulated during karyogamy.** (A) Schematic diagram of karyogamic progression during early zygotic development. Pink, green, and orange circles indicate the egg, sperm, and zygotic/embryonic nuclei, respectively. (B) Karyogamic progression in rice zygotes. A sperm cell expressing the H2B-GFP fusion protein was fused with a wild-type egg cell. (C) Two-celled embryo at 1 day after fusion. Top, middle, and bottom panels in B,C present fluorescent, merged fluorescent and brightfield, and brightfield images, respectively. Scale bars: 20 μm. (D-I) Scatter plots of log10(TPM+1) values for egg cells and zygotes. Red and blue dots represent upregulated and downregulated DEGs, respectively. N.S., transcripts not selected as DEGs (TPM>0 in both egg cells and zygotes at each stage). The number of transcripts is presented in parentheses.

The legend for Fig. 3 was incorrect. The corrected and original legends are shown below.

Corrected:

**Fig. 3. Initiation of *de novo* gene expression in the earliest zygote stage.** (A) Set visualization of upregulated DEGs in each zygote stage. Orange and green asterisks indicate persistent and stage-specific upregulation, respectively. (B) Transcripts exhibiting persistent upregulation extracted from the data shown in A. These categories were designated as P1-P5. (C) Heat maps for TPM values of P1 transcripts in sperm cells, egg cells, and early zygotes. Upper and lower clades indicate possible *de novo* expression and sperm cell-enriched transcription, respectively. (D) Transcripts exhibiting stage-specific upregulation extracted from the data shown in A. These categories were designated as S1-S6. (E) Heat maps for TPM values of S2 transcripts in sperm cells, egg cells, and early zygotes. (F,G) Expression patterns of representative P1 (F) and S2 (G) transcripts confirmed by semi-quantitative RT-PCR. Ubiquitin was used as an internal control. Numbers in parentheses indicate the number of PCR cycles.

Original:

**Fig. 3. Initiation of *de novo* gene expression in the earliest zygote stage.** (A) Set visualization of upregulated DEGs in each zygote stage. Orange and green asterisks indicate persistent and stage-specific upregulation, respectively. (B) Transcripts exhibiting persistent upregulation extracted from the data shown in A. These categories were designated as P1-P5. (C) Heat maps for TPM values of P1 transcripts in sperm cells, egg cells, and early zygotes. Upper and lower clades indicate possible *de novo* expression and sperm cell-enriched transcription, respectively. (D) Transcripts exhibiting stage-specific upregulation extracted from the data shown in A. These categories were designated as S1-S5. (E) Heat maps for TPM values of S2 transcripts in sperm cells, egg cells, and early zygotes. (F,G) Expression patterns of representative P1 (F) and S2 (G) transcripts confirmed by semi-quantitative RT-PCR. Ubiquitin was used as an internal control. Numbers in parentheses indicate the number of PCR cycles.

The online PDF has been corrected.

The authors apologise for these errors, which do not impact the results and conclusions of the paper.

