# Supplementary material for: Transcriptional dynamics during karyogamy in rice zygotes
Source: Development. 2025 Jan 27;152(2):DEV204497. doi: 10.1242/dev.204497 (PMC11829756; doi:10.1242/dev.204497)
Supplement: Supplementary information [file develop-152-204497-s1.pdf]

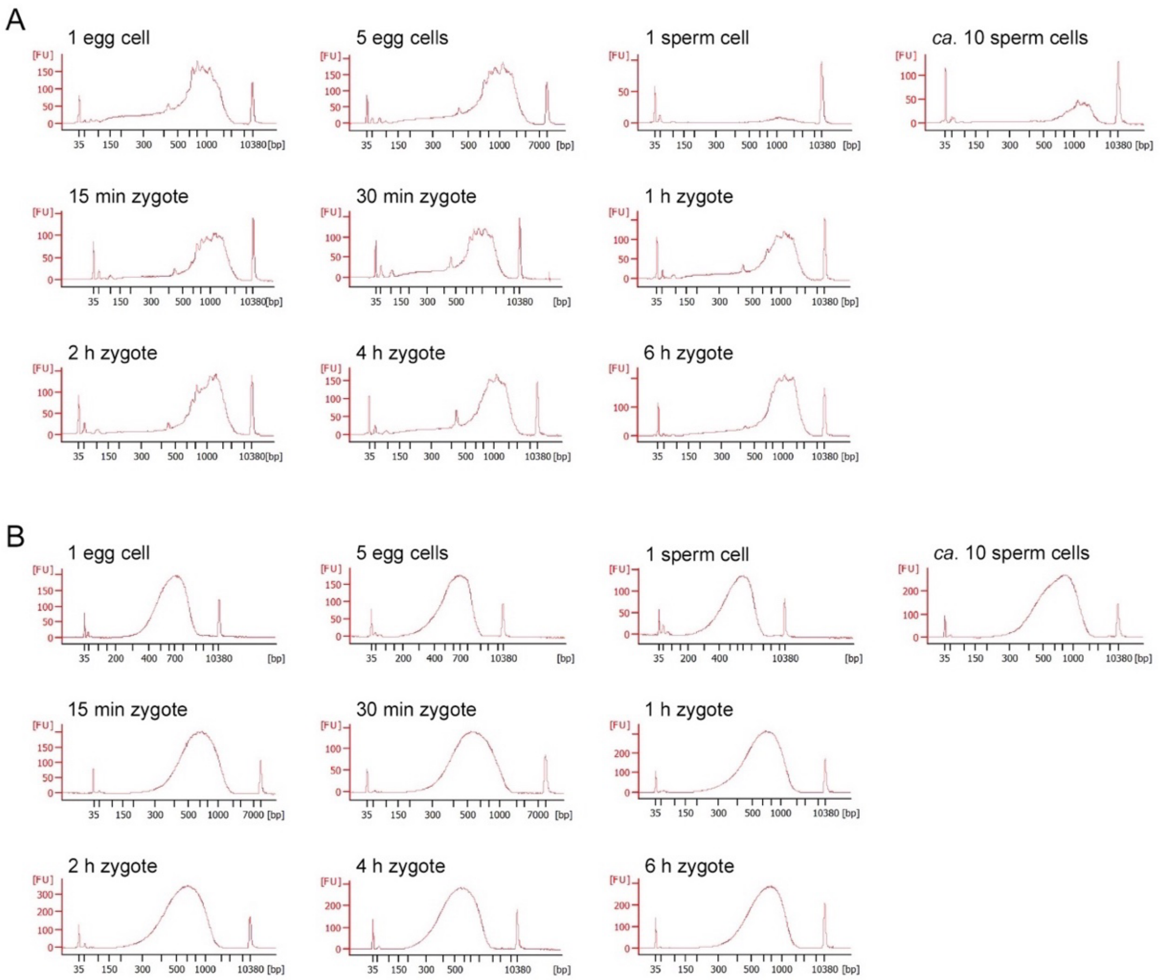

Fig. S1. Electropherograms of cDNAs and libraries for rice gametes and zygotes.

Synthesized and amplified cDNAs (A) and prepared libraries (B) were analyzed using the Agilent 2100 Bioanalyzer with a High Sensitivity DNA chip. FU, fluorescence absorption units.

Development • Supplementary information

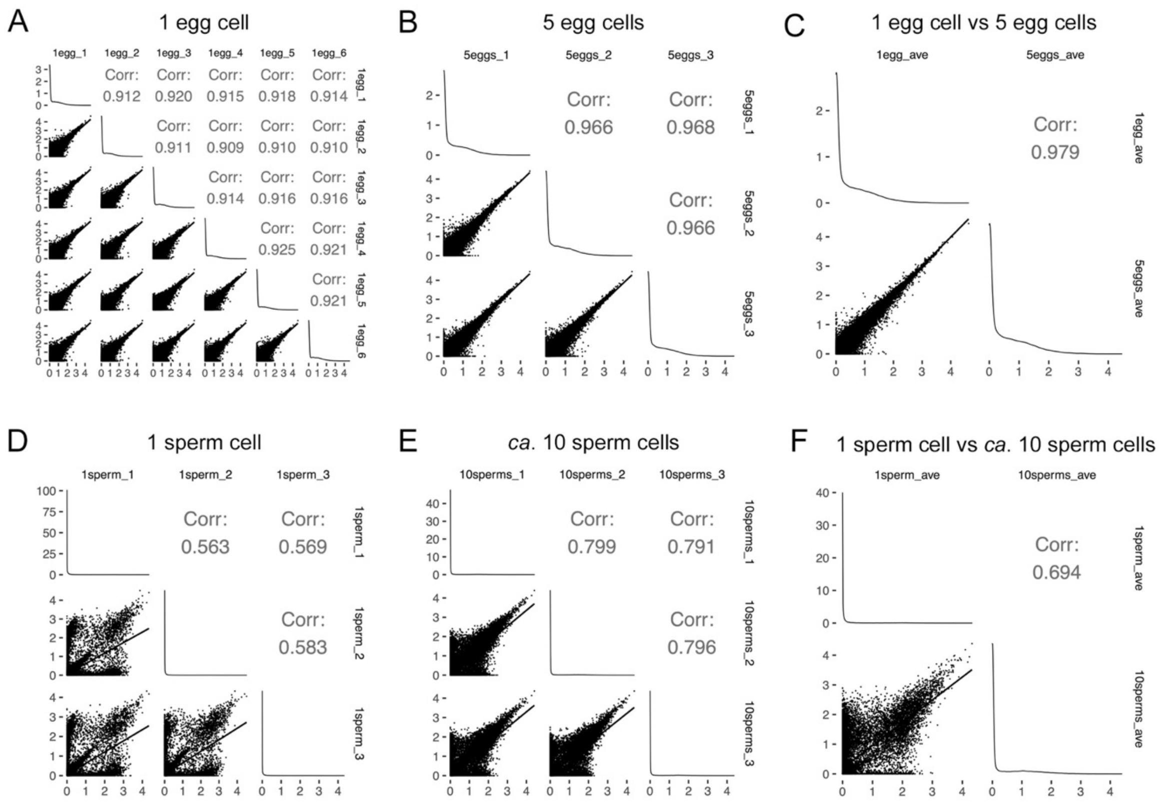

Fig. S2. Plots of TPM values with log conversion in single gametes.

(A, B) Scatter plots of log10[TPM + 1] values for sample replicates (A, one egg cell, six replicates; B, five egg cells, three replicates). (C) Scatter plots of average log10[TPM + 1] values for samples comprising one egg cell and five egg cells. (D, E) Scatter plots of log10[TPM + 1] values for sample replicates (D, one sperm cell, three replicates; E, approximately 10 sperm cells, three replicates). (F) Scatter plots of average log10[TPM + 1] values for samples consisting of one sperm cell and approximately 10 sperm cells. The numbers in the insets (i.e., Corr:) represent the Pearson correlation coefficient ($p < 0.001$). Black lines in plots indicate a linear regression.

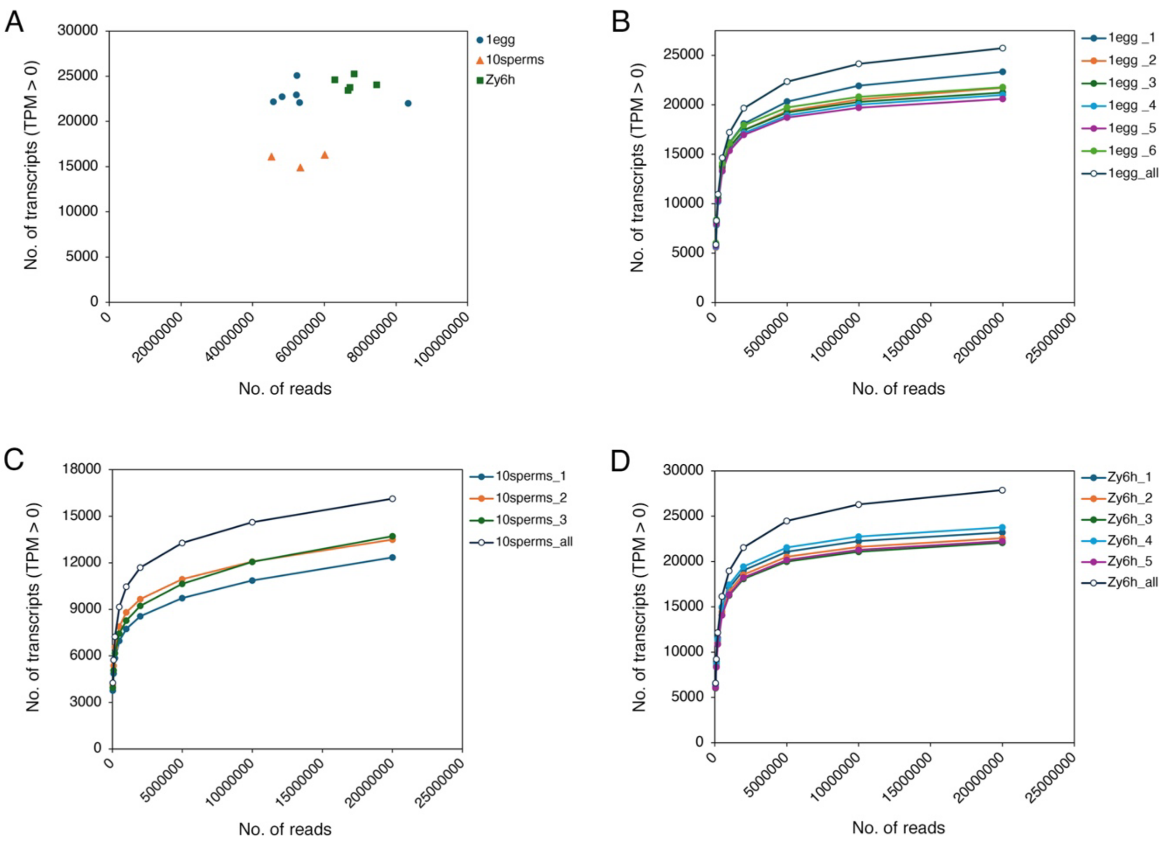

Fig. S3. Relationship between the number of RNA-seq reads and the number of expressed genes. (A) Plots of the number of transcripts detected from pre-processed reads in samples of egg cells, sperm cells and zygotes at 6 h after gamete fusion. (B–D) Plots of the number of transcripts detected from downsampled reads in samples of egg cells (B), sperm cells (C), and zygotes at 6 h after gamete fusion (D). Pre-processed reads were randomly downsampled to 20 million, 10 million, 5 million, 2 million, 1 million, 500K, 200K, 100K, and 50K. In addition, pre-processed reads of each sample replicate was pooled, and the combined reads were also analyzed after downsampling as above.

Development • Supplementary information

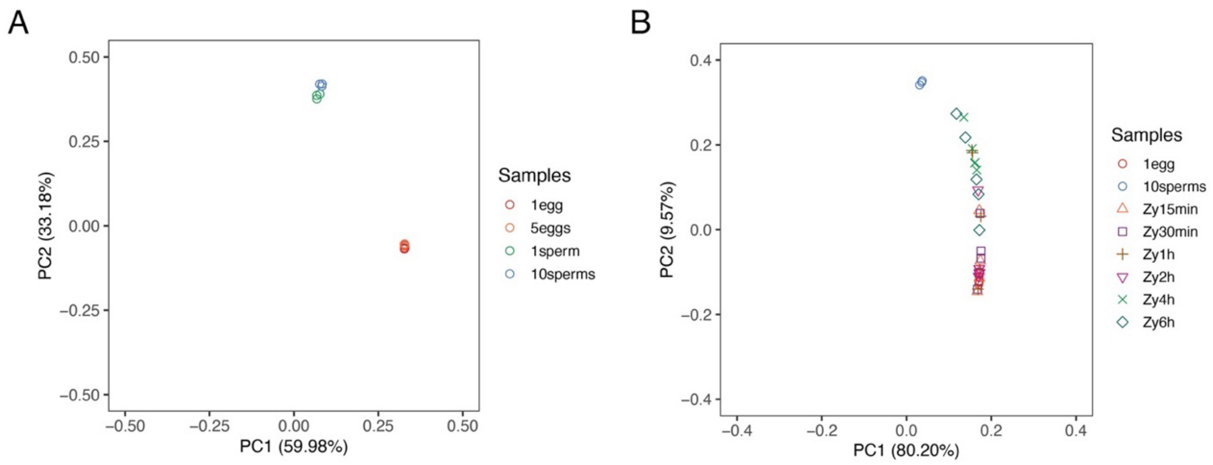

Fig. S4. Principal component analysis (PCA) of RNA-seq data for rice gametes and zygotes. PCA was performed using the prcomp function in R. TPM values were scaled and centered before the analysis. (A) PCA of TPM values for gametes. The first component (PC1) and the second component (PC2) separates the plots of the samples of egg cells and sperm cells. (B) PCA of TPM values for gametes and zygotes. PC2 mainly separates the plots of the samples of egg cells, sperm cells, and each stage of zygotes. Percentages in parentheses indicate the proportion of variances. Sample replicates are marked with the same colors and shapes for identification.

Development • Supplementary information

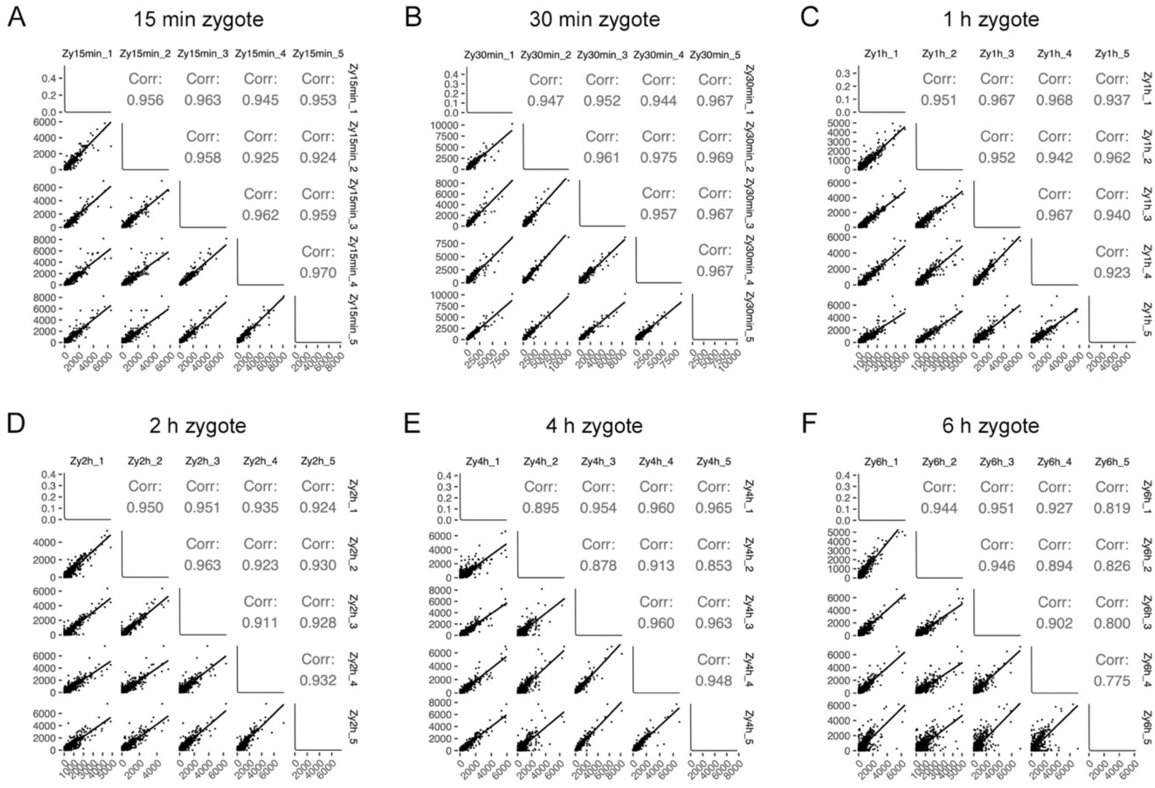

Fig. S5. Reliability of RNA-seq data for single zygotes in rice.

Scatter plots of TPM values for sample replicates of isogenic zygotes at each developmental stage, 15 min (A), 30 min (B), 1 h (C), 2 h (D), 4 h (E), and 6 h (F) after gamete fusion. The numbers in the insets (i.e., Corr:) represent the Pearson correlation coefficient ($p < 0.001$). The correlation coefficient was calculated after manually excluding clear outliers in the data. The numbers of the excluded transcripts as outliers are as follows: (A) 2, (B) 1, (C) 2, (D) 2, (E) 1, and (F) 1. Black lines in plots indicate a linear regression.

Development • Supplementary information

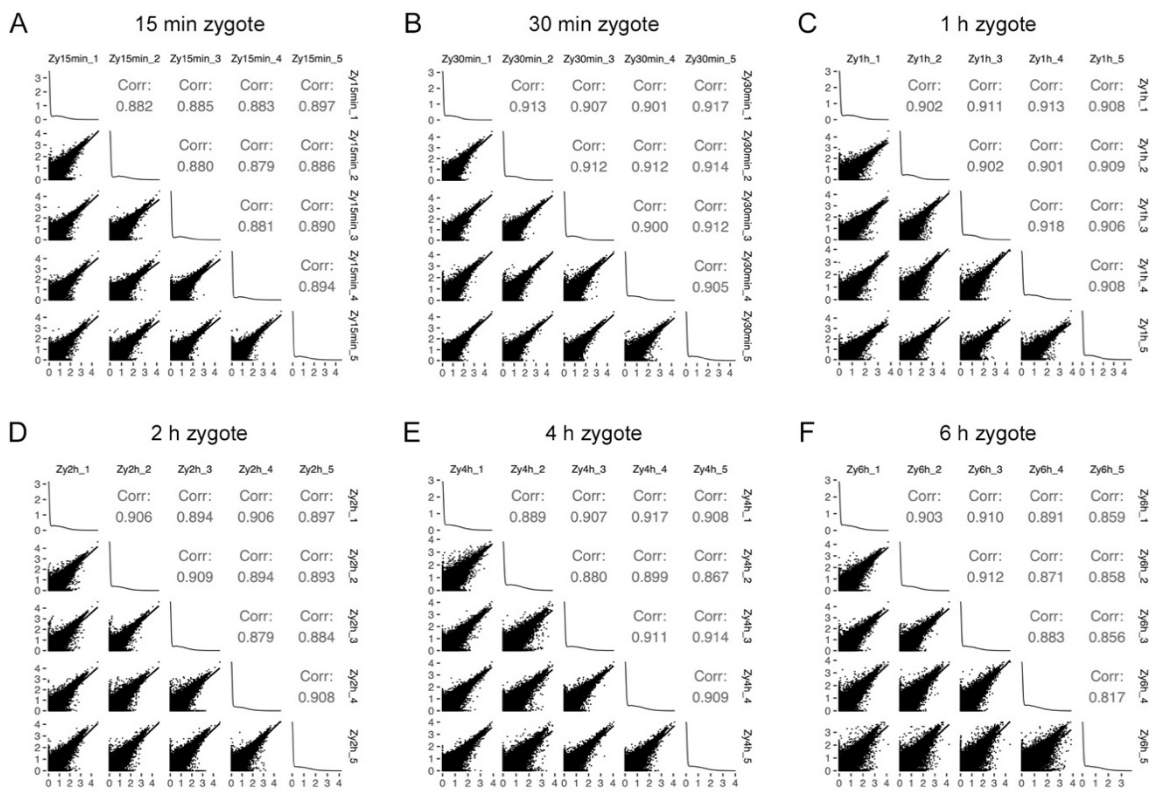

Fig. S6. Plots of TPM values with log conversion in single zygotes.

Scatter plots of log10[TPM + 1] values for sample replicates of isogenic zygotes at each developmental stage, 15 min (A), 30 min (B), 1 h (C), 2 h (D), 4 h (E), and 6 h (F) after gamete fusion. The numbers in the insets (i.e., Corr:) represent the Pearson correlation coefficient ($p < 0.001$). Black lines in plots indicate a linear regression.

Development • Supplementary information

A

Gene symbol	Average TPM in gametes and zygotes at each developmental stage							
	Sperm cell	Egg cell	15 min zygote	30 min zygote	1 h zygote	2 h zygote	4 h zygote	6 h zygote
OsFEB1	85.29	41803.56	34926.76	33318.31	23344.36	35442.25	9094.45	12807.53
OsFEB2	12.81	3139.15	2628.67	3594.08	3104.64	2389.93	762.56	1279.32
OsGCS1	2641.00	34.16	48.00	53.53	38.69	24.54	24.05	14.50
OsGCS1-like	236.59	1.68	4.26	3.20	1.10	1.39	1.51	2.88
OsGEX1	918.73	20.95	20.69	54.46	53.40	60.60	42.94	20.77
OsASGR-BBML1	0.01	0.02	0	0	0.81	10.47	20.16	22.33

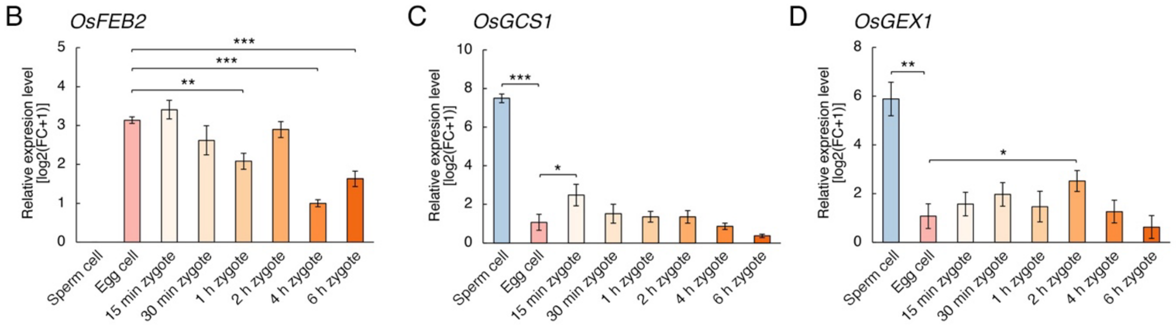

Fig. S7. Expression levels of genes involved in fertilization and subsequent development.

(A) TPM values of transcripts involved in fertilization and subsequent development. (B–D) RT-qPCR of *OsFEB2* (B), *OsGCS1* (C), and *OsGEX1* (D) in sperm cells, egg cells, and early zygotes. Expression level is represented relative to that in zygotes at 4 h after gamete fusion (B) and egg cells (C and D). Data represent mean ± SE, calculated from three independent biological and technical replicates. P.values were calculated using the two-tailed Student's t-test: $*p < 0.05$, $**p < 0.01$, $***p < 0.001$.

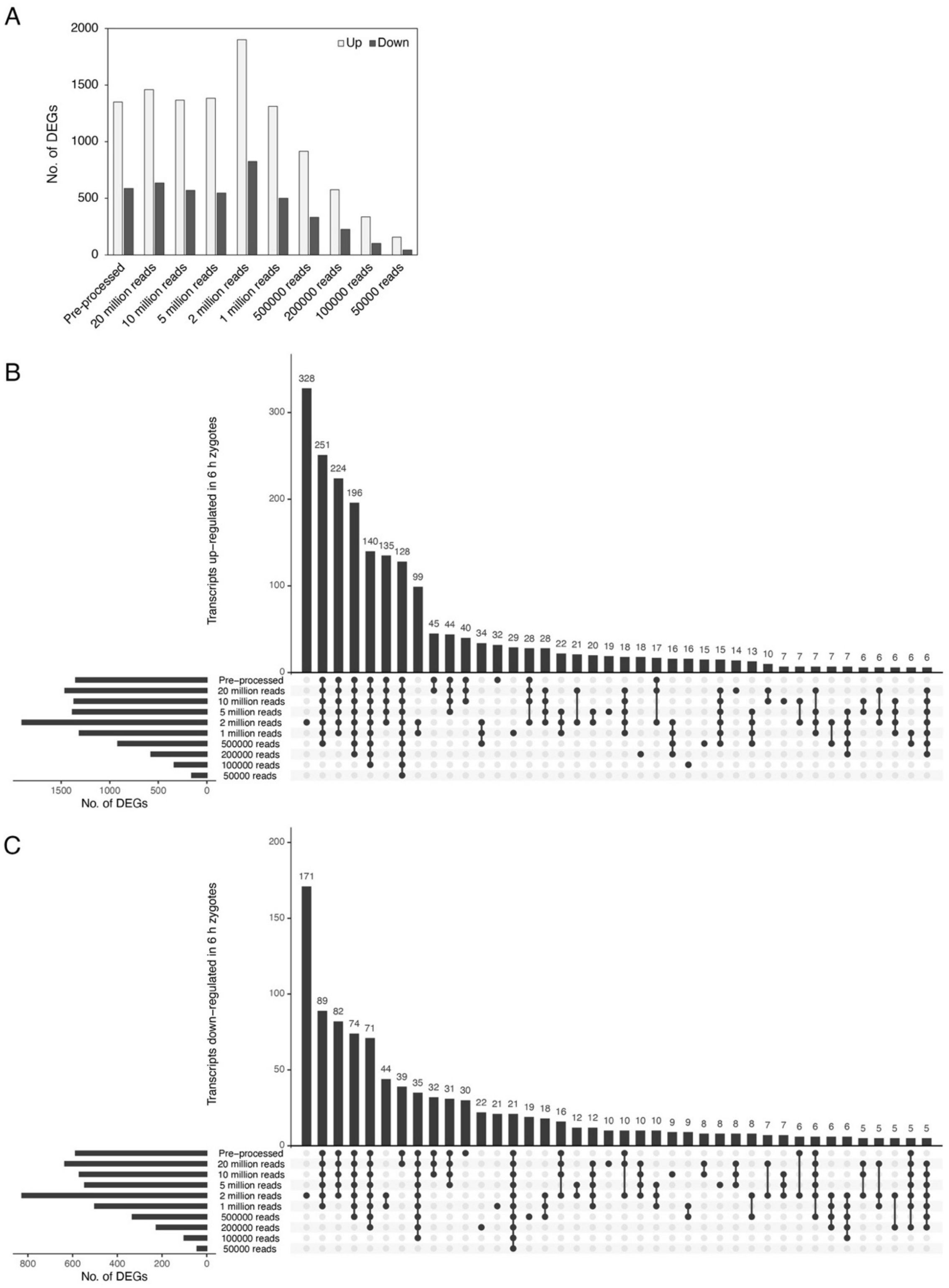

Fig. S8. Relationship between the number of RNA-seq reads and the resulting DEGs.
(A) The number of DEGs up- and down-regulated in zygotes at 6 h after gamete fusion (compared with egg cells) were calculated in pre-processed and downsampled reads. (B) Set visualization of upregulated DEGs in each sample size. (C) Set visualization of downregulated DEGs in each sample size. The number and specific DEGs were largely stable over 5 million reads, suggesting that the sequencing depth in this study is in appropriate range for DEG analysis.

Development • Supplementary information

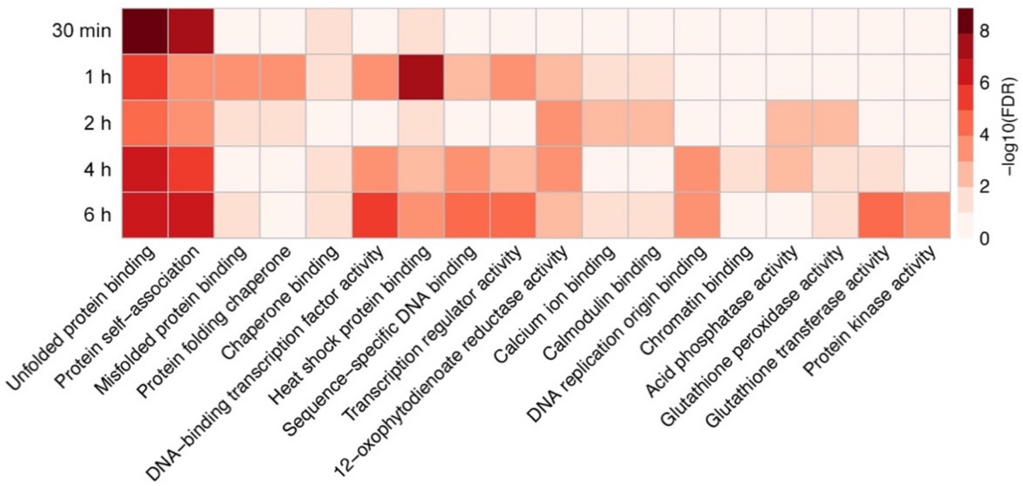

Fig. S9. Characteristic GO terms of the upregulated DEGs in early zygotes.

Characteristic GO terms (molecular function) of the upregulated DEGs in each zygote stage were calculated by ShinyGO (Ge *et al*., 2020). Representative top GO terms were represented.

Development • Supplementary information

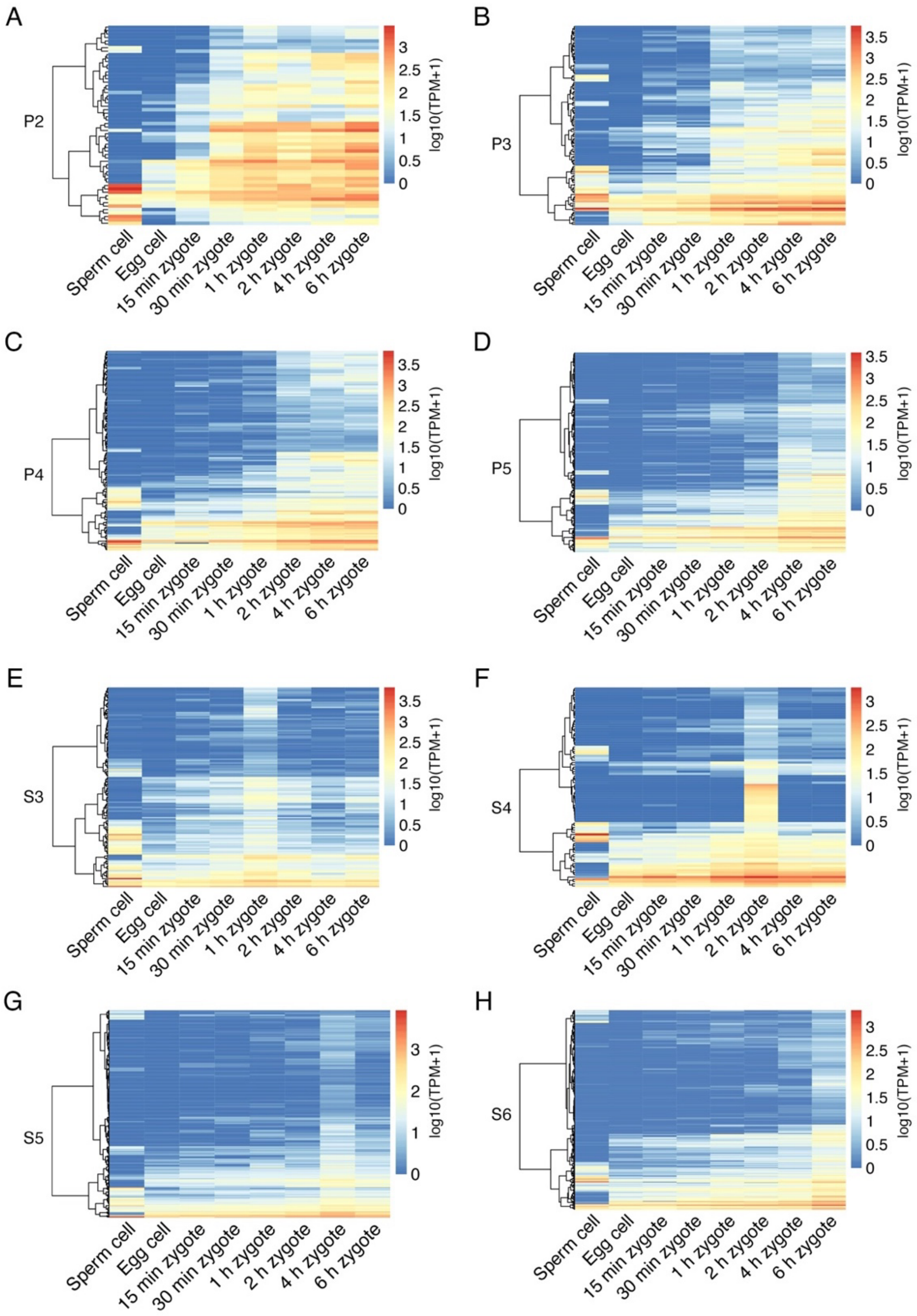

Fig. S10. Expression patterns of transcripts upregulated with persistent or stage-specific manner. Heat maps for TPM values of persistent upregulated transcripts, P2 (A), P3 (B), P4 (C), and P5 (D), and stage-specific upregulated transcripts, S3 (E), S4 (F), S5 (G), and S6 (H), in sperm cells, egg cells, and early zygotes.

Development • Supplementary information

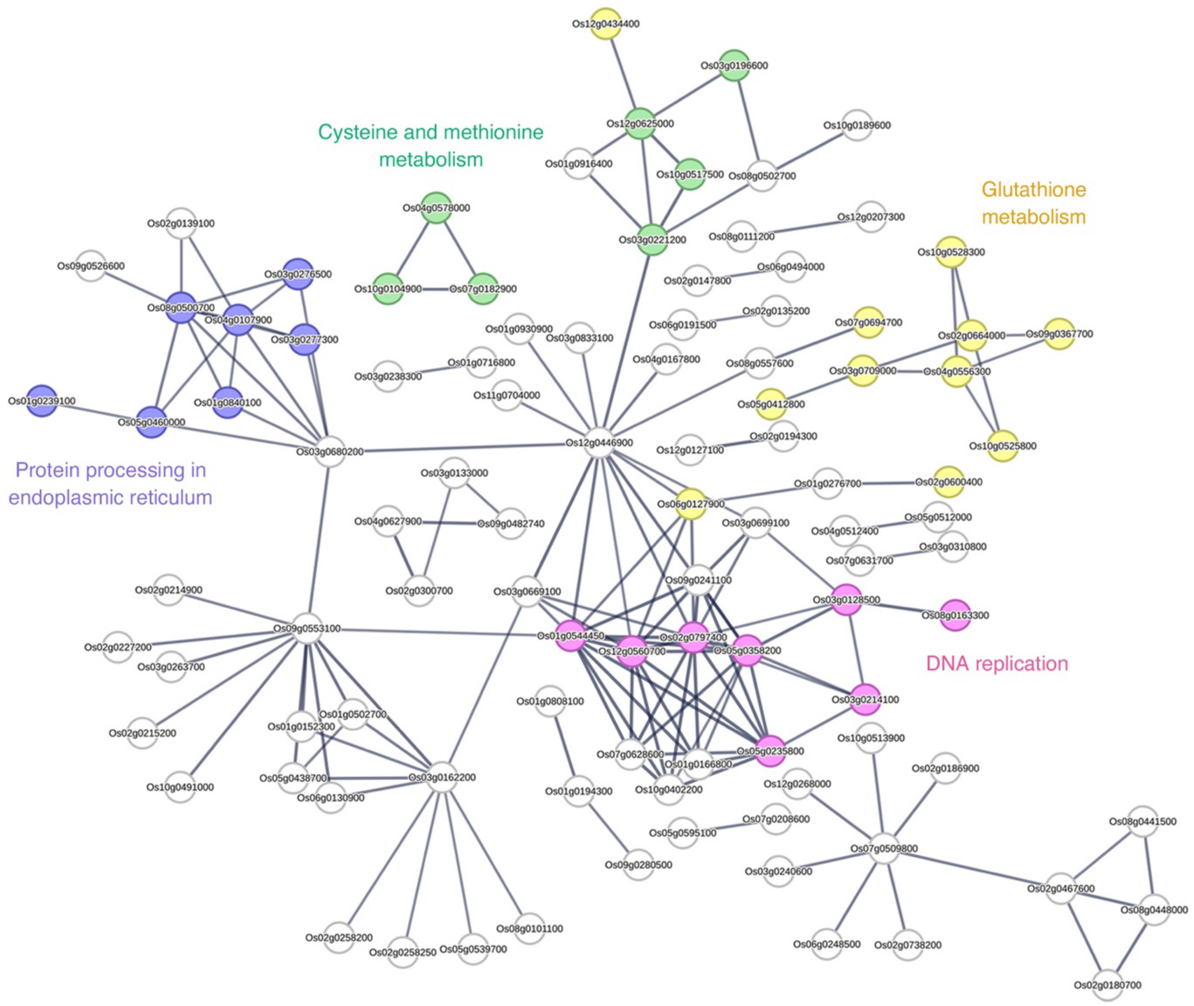

Fig. S11. Putative protein interactions among the upregulated transcripts in early zygotes. Multiple protein interactions in P1–P5 transcripts. Only connected nodes are represented in the network. The following KEGG pathways are indicated: 'Protein processing in endoplasmic reticulum' (purple), 'Glutathione metabolism' (yellow), 'Cysteine and methionine metabolism' (green), and 'DNA replication' (pink). There were significantly more protein interactions associated with this network than a similarly sized network encoding a random set of proteins (PPI enrichment $p < 1.0e\text{-}16$).

Development • Supplementary information

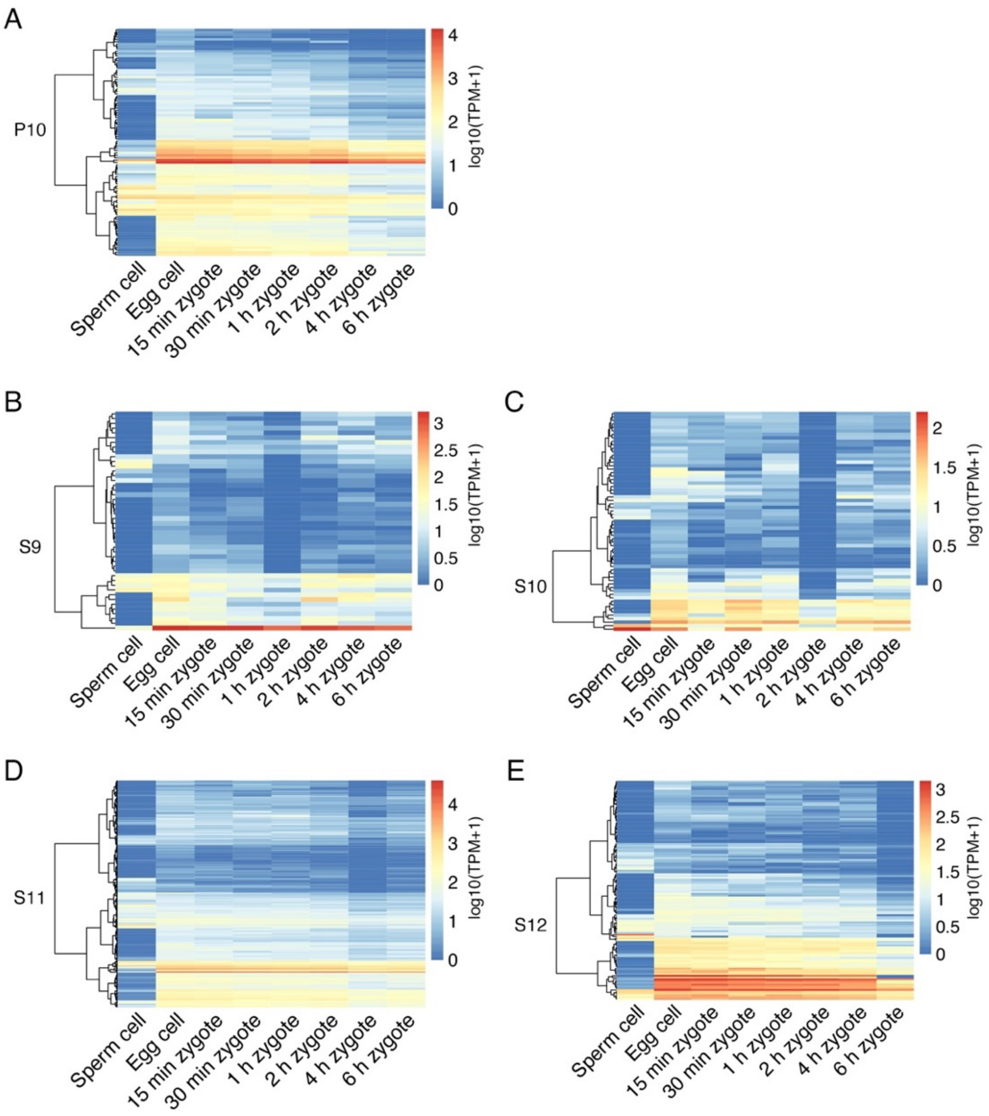

Fig. S12. Expression patterns of transcripts downregulated with persistent or stage-specific manner. Heat maps for TPM values of persistent downregulated transcripts, P10 (A), and stage-specific downregulated transcripts, S9 (B), S10 (C), S11 (D), and S12 (E), in sperm cells, egg cells, and early zygotes.

Development • Supplementary information

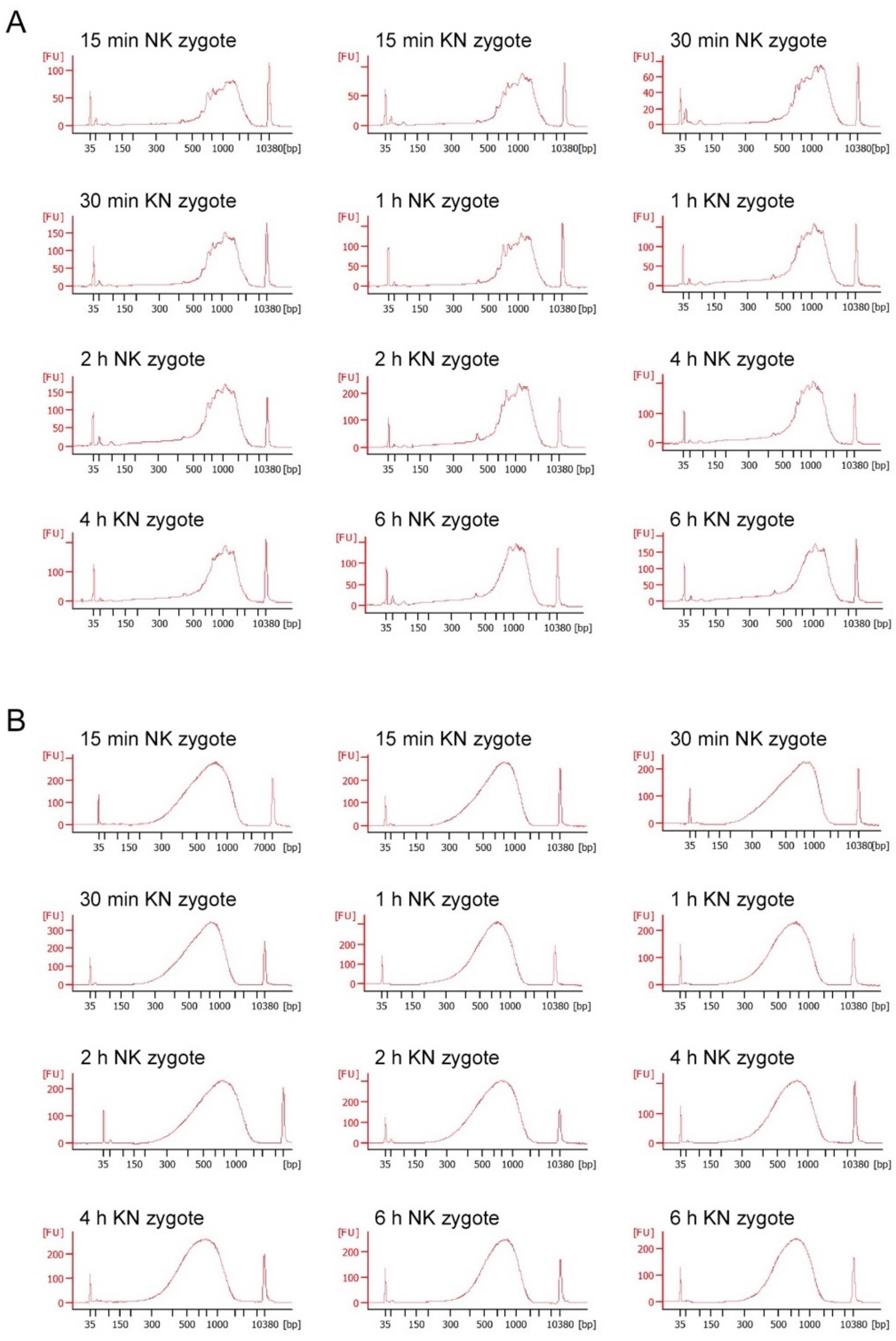

Fig. S13. Electropherograms of cDNA and libraries for rice intersubspecific zygotes. Synthesized and amplified cDNAs (A) and prepared libraries (B) were analyzed using the Agilent 2100 Bioanalyzer with a High Sensitivity DNA chip. FU, fluorescence absorption units.

Development • Supplementary information

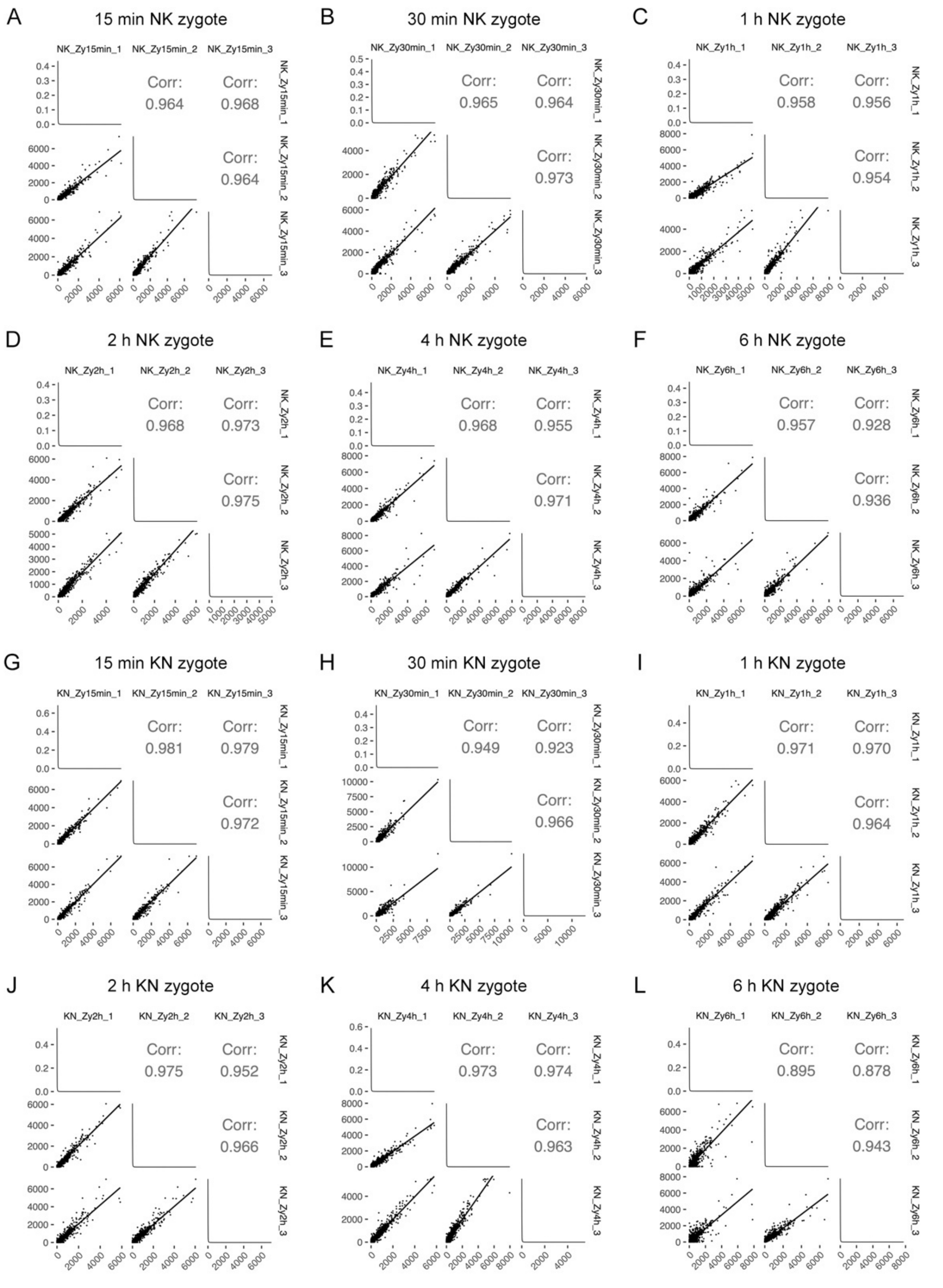

Fig. S14. Reliability of RNA-seq data for single intersubspecific zygotes in rice.
Scatter plots of TPM values for sample replicates of intersubspecific zygotes at each developmental stage, 15 min (A, G), 30 min (B, H), 1 h (C, I), 2 h (D, J), 4 h (E, K), and 6 h (F, L) after gamete fusion. The numbers in the insets (i.e., Corr:) represent the Pearson correlation coefficient ($p < 0.001$). The correlation coefficient was calculated after manually excluding clear outliers in the data. The numbers of the excluded transcripts as outliers are as follows: (A) 2, (B) 2, (C) 2, (D) 2, (E) 1, (F) 0, (G) 3, (H) 2, (I) 2, (J) 3, (K) 2, and (L) 2. Black lines in plots indicate a linear regression.

Development • Supplementary information

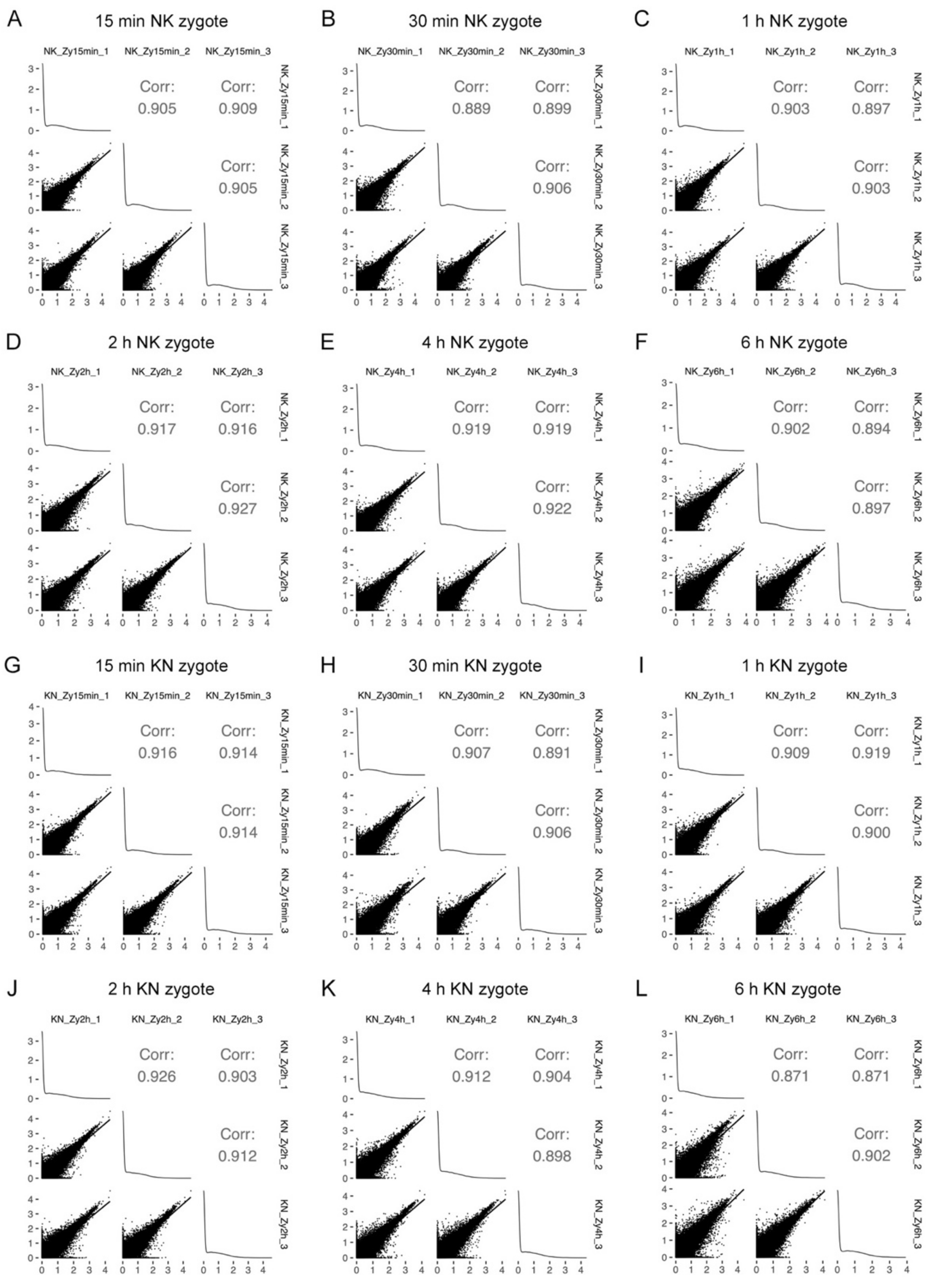

Fig. S15. Plots of TPM values with log conversion in single intersubspecific zygotes.

Scatter plots of log10[TPM + 1] values for sample replicates of intersubspecific zygotes at each developmental stage, 15 min (A, G), 30 min (B, H), 1 h (C, I), 2 h (D, J), 4 h (E, K), and 6 h (F, L) after gamete fusion. The numbers in the insets (i.e., Corr:) represent the Pearson correlation coefficient ($p < 0.001$). Black lines in plots indicate a linear regression.

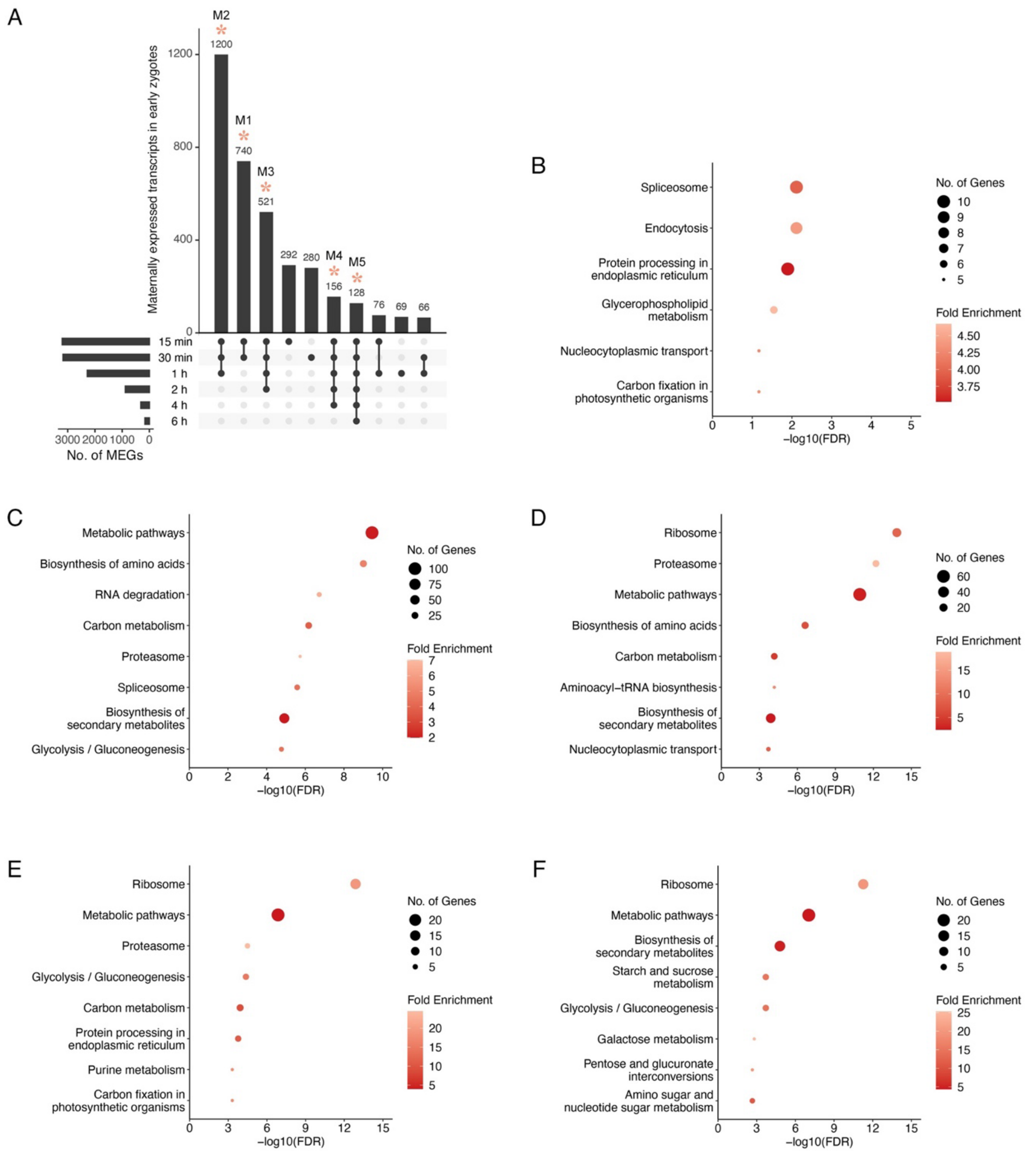

Fig. S16. Characterization of maternal-biased transcripts in early zygotes.

(A) Set visualization of MEGs in each zygote stage. Red asterisks indicate transcript categories with persistent maternal dependency. These categories were designated as M1–M5. (B–F) Characteristic KEGG pathways in transcript categories M1 (B), M2 (C), M3 (D), M4 (E), and M5 (F). Top KEGG pathways were represented for each transcript category.

Development • Supplementary information

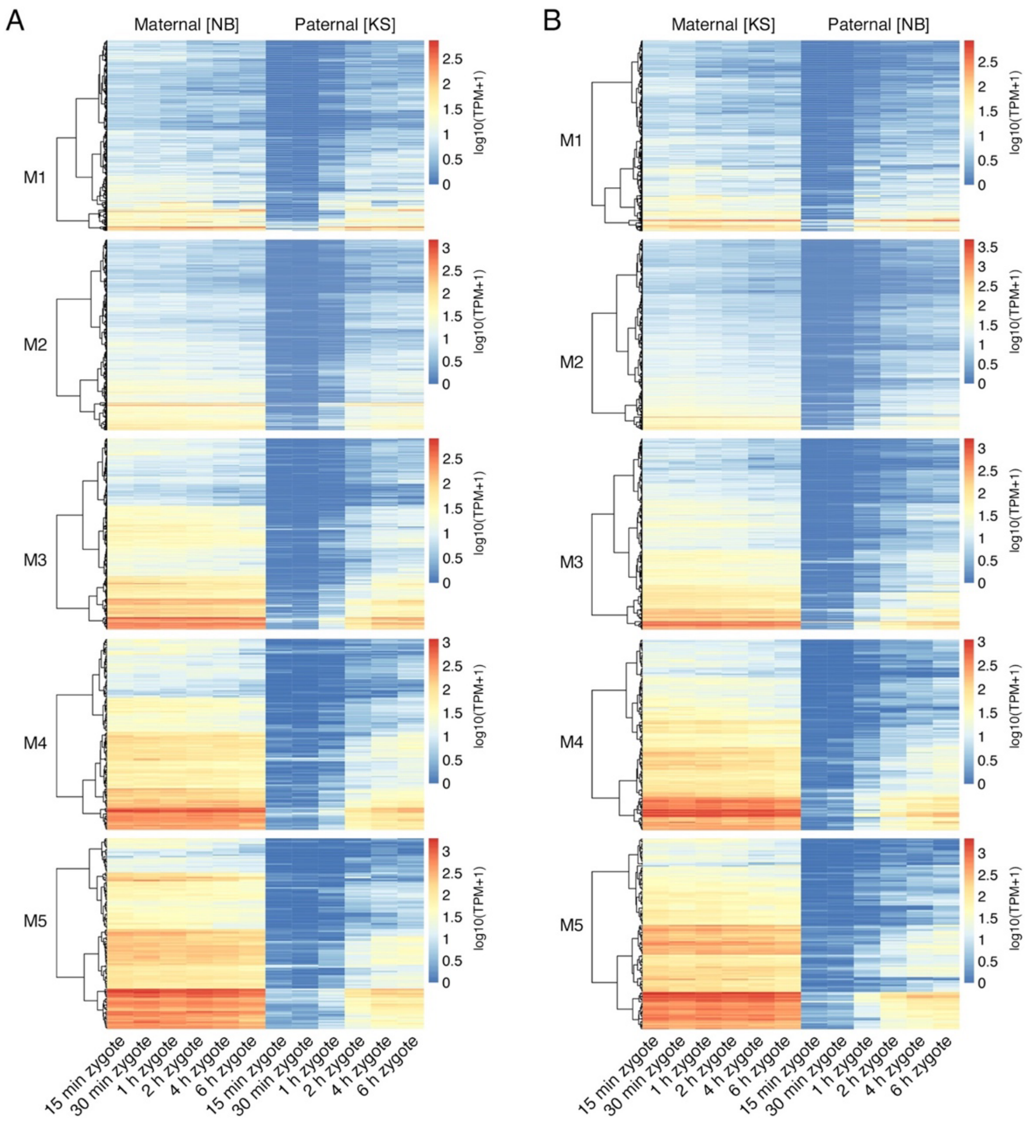

Fig. S17. Allelic expression patterns of persistent MEGs in early zygotes.

Heat maps for allelic TPM values of M1–M5 transcripts in NK zygotes (A) and KN zygotes (B) at 15 min–6 h after ganete fusion.

Development • Supplementary information

A

Transcript ID	Average TPM in gametes and zygotes at each developmental stage							
	Sperm cell	Egg cell	15 min zygote	30 min zygote	1 h zygote	2 h zygote	4 h zygote	6 h zygote
Os01t0841700-01	0	0	1.09	0.01	15.92[a]	39.57[a,b]	162.29[a,b]	443.76[a,b]
Os01t0895600-01	0.01	1.66	46.01	7.33	29.97	169.35[a,b]	835.45[a,b]	327.09[a,b]
Os02t0462800-01	21.61	0.08	13.83	23.56[a,b]	96.25[a,b]	54.96[a,b]	18.51[a,b]	55.00[a,b]
Os03t0214100-01	20.78	1.15	0.08	11.33	12.41	26.28	38.51[a]	87.11[a]
Os05t0571200-01	0	0	0	2.58	0.16	8.01[a]	100.43[a,b]	44.12[a,b]

a, FDR < 0.05 (TCC; Sun *et al.*, 2013); b, FDR < 0.05 (samr; Li and Tibshirani, 2013)

B

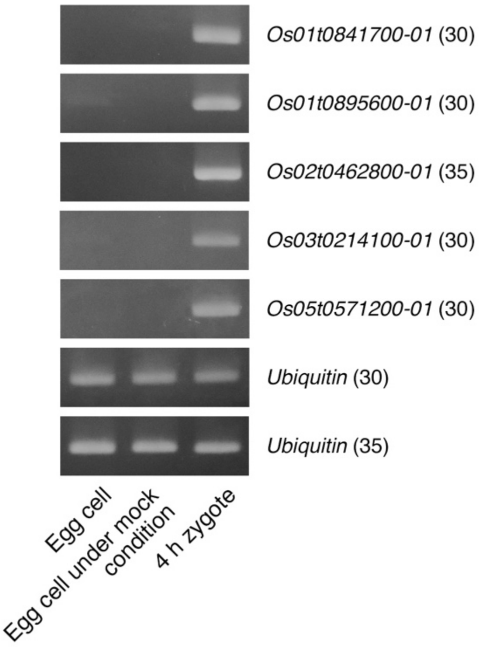

C

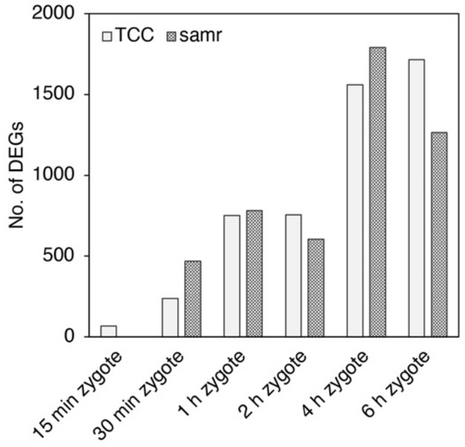

Fig. S18. Validation of appropriateness of the methods for identifying DEGs.

(A) TPM values of this study for fertilization-induced transcripts identified in rice zygotes (Abiko *et al.*, 2013). DEGs upregulated in zygotes at each stage (compared to egg cells) were calculated using TCC (Sun *et al.*, 2013) and samr (Li and Tibshirani, 2013), respectively. (B) Expression patterns of the fertilization-induced transcripts confirmed by semi-quantitative RT-PCR. Three egg cells were subjected to pseudo-electrofusion without sperm cells and incubated for 4 h. The egg cells under mock condition were sampled individually for cDNA synthesis as in egg cells and zygotes. Ubiquitin was used as an internal control. Numbers in parentheses indicate the number of PCR cycles. (C) Comparison of the number of DEGs calculated by TCC and samr methods. DEGs were calculated between egg cells and each stage of zygotes. FDR < 0.05 in both TCC and samr.

Table S1. Sample information for RNA-seq analysis.

Samples	Cultivars or Cross patterns (Egg cell × Sperm cell)	No. of cells	Replicates
1 egg cell	NB	1	6
5 egg cells	NB	5	3
1 sperm cell	NB	1	3
ca. 10 sperm cells	NB	11–13	3
15 min zygote	NB × NB	1	5
30 min zygote	NB × NB	1	5
1 h zygote	NB × NB	1	5
2 h zygote	NB × NB	1	5
4 h zygote	NB × NB	1	5
6 h zygote	NB × NB	1	5
15 min zygote	NB × KS	1	3
	KS × NB	1	3
30 min zygote	NB × KS	1	3
	KS × NB	1	3
1 h zygote	NB × KS	1	3
	KS × NB	1	3
2 h zygote	NB × KS	1	3
	KS × NB	1	3
4 h zygote	NB × KS	1	3
	KS × NB	1	3
6 h zygote	NB × KS	1	3
	KS × NB	1	3

Table S2. Total reads of gamete samples.

Samples	Total read bases (bp)	Total reads
1 egg cell_1	8,822,483,342	58,427,042
1 egg cell_2	8,659,255,362	57,346,062
1 egg cell_3	7,639,874,898	50,595,198
1 egg cell_4	8,770,242,174	58,081,074
1 egg cell_5	13,614,994,426	90,165,526
1 egg cell_6	7,881,859,344	52,197,744
5 egg cells_1	10,844,418,038	71,817,338
5 egg cells_2	8,118,221,456	53,763,056
5 egg cells_3	7,817,412,544	51,770,944
1 sperm cell_1	13,794,278,538	91,352,838
1 sperm cell_2	13,817,552,168	91,506,968
1 sperm cell_3	10,556,792,634	69,912,534
ca. 10 sperm cells_1	8,391,708,730	55,574,230
ca. 10 sperm cells_2	9,461,545,240	62,659,240
ca. 10 sperm cells_3	7,151,495,296	47,360,896

Development • Supplementary information

Table S3. Mapping rate of transcript sequences.

Samples	Mapping rate (%)						
	Rep_1	Rep_2	Rep_3	Rep_4	Rep_5	Rep_6	Average
1 egg cell	66.87	67.02	65.80	71.31	72.40	70.20	68.93
5 egg cells	69.91	72.12	73.23	-	-	-	71.75
1 sperm cell	47.28	60.36	54.47	-	-	-	54.05
ca. 10 sperm cells	69.93	69.66	68.88	-	-	-	69.49
15 min zygote	72.01	73.65	72.69	73.86	73.70	-	73.18
30 min zygote	68.22	70.75	70.15	71.71	70.57	-	70.28
1 h zygote	71.75	70.27	71.73	73.03	70.94	-	71.54
2 h zygote	74.33	73.14	65.27	71.52	70.03	-	70.86
4 h zygote	71.86	71.14	72.72	72.92	71.90	-	72.11
6 h zygote	73.95	67.33	70.67	71.89	76.23	-	72.01
15 min NK zygote	75.06	74.79	74.40	-	-	-	74.75
15 min KN zygote	71.44	72.35	71.69	-	-	-	71.83
30 min NK zygote	73.72	75.69	75.39	-	-	-	74.93
30 min KN zygote	72.06	72.86	73.51	-	-	-	72.81
1 h NK zygote	74.61	75.16	74.58	-	-	-	74.78
1 h KN zygote	69.03	67.84	70.96	-	-	-	69.28
2 h NK zygote	72.81	72.53	72.92	-	-	-	72.75
2 h KN zygote	70.48	69.75	67.28	-	-	-	69.17
4 h NK zygote	72.51	71.56	72.56	-	-	-	72.21
4 h KN zygote	71.75	71.88	68.69	-	-	-	70.77
6 h NK zygote	71.99	71.87	72.20	-	-	-	72.02
6 h KN zygote	68.90	70.93	71.42	-	-	-	70.42

Development • Supplementary information

Table S4. Total reads of isogenic zygote samples.

Samples	Total read bases (bp)	Total reads
15 min zygote_1	7,484,115,680	49,563,680
15 min zygote_2	6,813,323,548	45,121,348
15 min zygote_3	7,751,987,264	51,337,664
15 min zygote_4	7,578,131,904	50,186,304
15 min zygote_5	7,747,983,952	51,311,152
30 min zygote_1	8,253,937,236	54,661,836
30 min zygote_2	11,126,210,312	73,683,512
30 min zygote_3	9,113,229,312	60,352,512
30 min zygote_4	8,145,017,010	53,940,510
30 min zygote_5	8,381,254,094	55,504,994
1 h zygote_1	7,219,703,808	47,812,608
1 h zygote_2	6,582,559,006	43,593,106
1 h zygote_3	8,183,393,962	54,194,662
1 h zygote_4	7,878,515,298	52,175,598
1 h zygote_5	6,699,931,608	44,370,408
2 h zygote_1	9,304,787,006	61,621,106
2 h zygote_2	7,292,130,354	48,292,254
2 h zygote_3	8,384,415,128	55,525,928
2 h zygote_4	7,901,124,830	52,325,330
2 h zygote_5	8,163,436,896	54,062,496
4 h zygote_1	10,813,940,802	71,615,502
4 h zygote_2	9,442,561,822	62,533,522
4 h zygote_3	9,892,820,870	65,515,370
4 h zygote_4	10,168,742,264	67,342,664
4 h zygote_5	9,032,879,494	59,820,394
6 h zygote_1	9,978,686,718	66,084,018
6 h zygote_2	11,995,785,790	79,442,290
6 h zygote_3	10,722,847,032	71,012,232
6 h zygote_4	10,865,853,998	71,959,298
6 h zygote_5	10,647,926,570	70,516,070

Development • Supplementary information

Table S5. Expression profiles of upregulated DEGs during karyogamy in rice zygotes.

Available for download at
https://journals.biologists.com/dev/article-lookup/doi/10.1242/dev.204497#supplementary-data

Table S6. Expression profiles of downregulated DEGs during karyogamy in rice zygotes.

Available for download at
https://journals.biologists.com/dev/article-lookup/doi/10.1242/dev.204497#supplementary-data

Table S7. Expression profiles of transcripts upregulated with persistent or stage-specific manner.

Available for download at
https://journals.biologists.com/dev/article-lookup/doi/10.1242/dev.204497#supplementary-data

Table S8. Expression profiles of transcripts downregulated with persistent or stage-specific manner.

Available for download at
https://journals.biologists.com/dev/article-lookup/doi/10.1242/dev.204497#supplementary-data

Table S9. Total reads of intersubspecific zygote samples.

Samples	Cross patterns (Egg cell × Sperm cell)	Total read bases (bp)	Total reads
15 min zygote NK_1	NB × KS	5,169,085,756	34,232,356
15 min zygote NK_2		7,643,760,732	50,620,932
15 min zygote NK_3		7,824,313,244	51,816,644
15 min zygote KN_1	KS × NB	7,081,862,854	46,899,754
15 min zygote KN_2		8,730,555,750	57,818,250
15 min zygote KN_3		7,432,328,720	49,220,720
30 min zygote NK_1	NB × KS	8,724,454,142	57,777,842
30 min zygote NK_2		7,941,494,680	52,592,680
30 min zygote NK_3		8,924,477,198	59,102,498
30 min zygote KN_1	KS × NB	8,181,221,374	54,180,274
30 min zygote KN_2		6,827,958,166	45,218,266
30 min zygote KN_3		7,352,302,948	48,690,748
1 h zygote NK_1	NB × KS	7,796,206,406	51,630,506
1 h zygote NK_2		7,944,974,626	52,615,726
1 h zygote NK_3		7,745,013,178	51,291,478
1 h zygote KN_1	KS × NB	8,022,244,648	53,127,448
1 h zygote KN_2		9,411,034,834	62,324,734
1 h zygote KN_3		9,046,295,542	59,909,242
2 h zygote NK_1	NB × KS	8,695,660,254	57,587,154
2 h zygote NK_2		8,131,157,626	53,848,726
2 h zygote NK_3		7,411,884,528	49,085,328
2 h zygote KN_1	KS × NB	7,427,079,054	49,185,954
2 h zygote KN_2		7,756,459,582	51,367,282
2 h zygote KN_3		6,983,158,382	46,246,082
4 h zygote NK_1	NB × KS	7,589,401,940	50,260,940
4 h zygote NK_2		7,617,248,152	50,445,352
4 h zygote NK_3		7,708,126,294	51,047,194
4 h zygote KN_1	KS × NB	8,253,806,470	54,660,970
4 h zygote KN_2		8,236,424,558	54,545,858
4 h zygote KN_3		6,826,146,166	45,206,266
6 h zygote NK_1	NB × KS	9,174,968,078	60,761,378
6 h zygote NK_2		8,472,833,178	56,111,478
6 h zygote NK_3		7,724,562,040	51,156,040
6 h zygote KN_1	KS × NB	8,912,441,290	59,022,790
6 h zygote KN_2		8,784,465,166	58,175,266
6 h zygote KN_3		8,103,821,794	53,667,694

Table S10. Origin of reads in intersubspecific zygote samples.

Origin of reads	Cross patterns (Egg cell × Sperm cell)	Samples	Maternal	Paternal	Common (unclassifiable)
All	NB × KS	15 min zygote	6,187,248	66,547	2,930,168
		30 min zygote	7,428,042	96,624	3,681,714
		1 h zygote	7,066,222	162,587	3,227,376
		2 h zygote	5,514,424	352,863	2,734,556
		4 h zygote	6,023,496	495,503	3,088,537
		6 h zygote	6,476,851	668,734	3,422,001
	KS × NB	15 min zygote	1,572,868	4,611,677	3,344,828
		30 min zygote	1,595,229	4,783,699	3,349,839
		1 h zygote	1,673,579	5,388,685	3,572,308
		2 h zygote	1,223,510	4,763,316	2,941,049
		4 h zygote	1,142,346	5,035,910	3,113,363
		6 h zygote	1,211,729	5,967,382	3,459,363
Comparable genes between NB and KS	NB × KS	15 min zygote	1,263,568	40,411	2,922,139
		30 min zygote	1,438,314	31,835	3,671,774
		1 h zygote	1,357,590	123,629	3,218,914
		2 h zygote	977,072	329,469	2,727,261
		4 h zygote	987,191	469,722	3,080,027
		6 h zygote	1,035,709	654,996	3,411,534
	KS × NB	15 min zygote	1,521,260	39,038	3,337,036
		30 min zygote	1,537,786	50,174	3,339,509
		1 h zygote	1,635,232	192,025	3,563,056
		2 h zygote	1,192,395	271,013	2,932,312
		4 h zygote	1,111,112	415,421	3,105,686
		6 h zygote	1,184,130	586,727	3,448,685

Development • Supplementary information

Table S11. Primers used for semi-quantitative RT-PCR and RT-qPCR.

Experiment	Target locus	Forward (5'–3')	Reverse (5'–3')
Semi-quantitative RT-PCR	*Os01g0384800*	GGCCAACAACCATCAGAGC	CGGCCTTCCTGAACGGGA
	Os01g0841700	AAGAGAAAGATGGCGGGGAG	GTGGTATGTTTTGTCCGCGA
	Os01g0895600	CCGACTCTTCCTCCGATTCA	TCCGCATATATCTGGCCCAA
	Os01g0955100	GTGCATGAAGACGACGCTC	CACCCTTCCCCTTCCATCTC
	Os02g0175100	ATGGACATCGAGGCGTTCAT	CCTGTGACTCGAGATCAGCT
	Os02g0462800	ATTATGCTTCTGATGGCCGC	AGTAGTTTGGTCAGTCGTCCT
	Os03g0214100	AAGACGATGCTTCTCACCCA	GACACGCACCTTGATGATCC
	Os03g0648500	TCGTGTTCAGGTTCGAGACG	ATTCTGGGAGGCGACGTAC
	Os04g0552300	TGAGTTAGCGTCGGATGTGT	ACGAGTTCTTGTAGTTCCGGT
	Os04g0684900	ACCTGGTCAAGCTCCTCATG	GAGGGTGTTTTGTTGCTGGG
	Os05g0571200	CCGCGTTCAAGAAGAGGAAG	TGCCACTACCTGCTGATGAA
	Os06g0162700	CACCCAGGGAATACTCACCA	AAGATTGTGTGCGTGTGACC
	Os06g0594400	GGGTTACAACGAGCTGATTCC	TGAGGAAGTCATACACGAGCA
	Os02g0161900 (Ubiquitin)	TCGTGAAGACCTTGACTGGG	TGGCTGATTACTGACCACCA
RT-qPCR	*Os05g0269500*	GCATCTAGTGTACGTGGGATTG	GGCAAACGTCAATGTCAAGAAC
	Os09g0442400	GCATAACACGGAGAGGCTTG	CTGACCTCTCCTCGATCACC
	Os11g0168000	CCGTCCTCCTACTACTTGCC	TCCAGCACTCCATCATCCAC
	Os02g0161900 (Ubiquitin)	GAGCCTCTGTTCGTCAAGTA	ACTCGATGGTCCATTAAACC
