# Supplementary material for: Transcriptional dynamics during karyogamy in rice zygotes
Source: Development. 2025 Jan 27;152(2):DEV204497. doi: 10.1242/dev.204497 (PMC11829756; doi:10.1242/dev.204497)
Supplement: Supplementary file 1 [file develop-152-204497_review_history.pdf]

Transcriptional dynamics during karyogamy in rice zygotes

Erika Toda, Shizuka Koshimizu, Atsuko Kinoshita, Tetsuya Higashiyama, Takeshi Izawa, Kentaro Yano and Takashi Okamoto
DOI: 10.1242/dev.204497

Editor: Dominique C Bergmann

Review timeline

Original submission:	14 May 2024
Editorial decision:	18 June 2024
First revision received:	31 October 2024
Editorial decision:	9 December 2024
Second revision received:	18 December 2024
Accepted:	18 December 2024

Original submission

First decision letter

MS ID#: DEVELOP/2024/203055

MS TITLE: Transcriptional dynamics during karyogamy in rice zygotes

AUTHORS: Erika Toda, Shizuka Koshimizu, Atsuko Kinoshita, Tetsuya Higashiyama, Takeshi Izawa, Kentaro Yano, Takashi Okamoto

I have now received all the referees' reports on the above manuscript, and have reached a decision. The referees' comments are appended below, or you can access them online: please go to BenchPress and click on the 'Manuscripts with Decisions' queue in the Author Area.

As you will see, the referees express considerable interest in your work, but have some significant criticisms and recommend a substantial revision of your manuscript before we can consider publication. If you are able to revise the manuscript along the lines suggested, which may involve further experiments, I will be happy receive a revised version of the manuscript. Specifically, the technical issues about the sequencing data raised by Rev 2 and Rev 3 must be addressed. In addition, the reviewers realize that the "in vitro" gamete fusion is the only way to ensure precise timing, but they worry whether the electrofusion may produce artifacts. Please provide additional data about the later development of the products of the fusion, and discuss this limitation in the text.

Your revised paper will be re-reviewed by one or more of the original referees, and acceptance of your manuscript will depend on your addressing satisfactorily the reviewers' major concerns. Please also note that Development will normally permit only one round of major revision. If it would be helpful, you are welcome to contact us to discuss your revision in greater detail. Please send us a point-by-point response indicating your plans for addressing the referees' comments, and we will look over this and provide further guidance.

Reviewer 1

Advance summary and potential significance to field

© 2025. Published by The Company of Biologists under the terms of the Creative Commons Attribution License (https://creativecommons.org/licenses/by/4.0/).

The manuscript entitled “Transcriptional dynamics during karyogamy in rice zygotes” reports transcript data during the nuclear fusion of male and female gametes and zygote development in rice. Although rice egg cell and zygote transcriptomes were already reported, the transcript data at specific time points before and after karyogamy are valuable information for understanding some critical developmental events on parental contributions to the zygote development and embryogenesis. The data analysis reported in this work is based on isolated rice zygotes and isolation procedure and zygote developmental in vitro condition for hours may modify the transcriptome, however, isolation of gametes and fusion of the gametes in vitro are the only way at moment to exactly control the time course of karyogamy. Dr. Okamoto’s lab is skillful in rice gamete isolation and in vitro fertilization. Therefore, the data are not perfect, but still very useful.

Comments for the author

The manuscript entitled “Transcriptional dynamics during karyogamy in rice zygotes” reports transcript data during the nuclear fusion of male and female gametes and zygote development in rice. Although rice egg cell and zygote transcriptomes were already reported, the transcript data at specific time points before and after karyogamy are valuable information for understanding some critical developmental events on parental contributions to the zygote development and embryogenesis. The data analysis reported in this work is based on isolated rice zygotes and isolation procedure and zygote developmental in vitro condition for hours may modify the transcriptome, however, isolation of gametes and fusion of the gametes in vitro are the only way at moment to exactly control the time course of karyogamy. Dr. Okamoto’s lab is skillful in rice gamete isolation and in vitro fertilization. Therefore, the data are not perfect, but still very useful.

The manuscript is generally well-written and data quality is acceptable.

Major concerns
1.In this work authors discussed “de novo and altered gene expression in zygotes from 15 min postfertilization”. According to Fig 2, at 15 min after gamete fusion the karyogamy is not yet initiated and thus zygote genome is not yet established. Generally, it is considered that the transcripts in zygote are either gamete-delivered during fertilization or de novo transcribed by zygote genome. If authors’ conclusion was true, then, there should be the third part of the transcripts, that is paternal genome or maternal genome transcription after fertilization and before nuclear fusion and zygote genome establishment.
This can be an important new finding or just a character of in vitro fertilization. Please carefully think about it and discuss this issue in the manuscript.
2.To confirm de novo transcription, authors used semi-quantitative RT-PCR, but detailed conditions for perform this experiment are not well described. This is important for readers to understand how the conclusion was made.
3.Authors mentioned that “paternal allelic expression was initiated from 1 h after gamete fusion” when male and female gamete nuclei underwent fusion.
Normally, paternal or maternal allelic expression is used to describe gene expression after zygotic genome activation. However, at 1 h after gamete fusion zygotic genome is not yet established, not to mention zygotic genome activation.
Therefore, this description may cause confusion.
4.According to Fig 2, 15-30 min after gamete fusion is very important period to reveal what happens before karyogamy and after gamete fusion. Up to now, almost nothing has been known about the mechanism underlying gamete nuclear fusion except some mutants interrupted this process. Adding a section to analyses how many and what type of genes expressed or upregulated in this period would be helpful for further investigations.

Minor concerns
1.In Summary statement: “in plant zygotes” can be deleted since rice zygote is already mentioned or move “rice zygote” to the end of the sentence.
2.P3, “implying the mixing of the male and female genomes before the first cell division”. The word “mixing” is not properly used here. Maybe, integrating is better.
3.P5, “Although karyogamy seems to be essential for proper embryogenesis”. Recently, several reports revealed that parthenogenesis is possible without karyogamy.

© 2025. Published by The Company of Biologists under the terms of the Creative Commons Attribution License (https://creativecommons.org/licenses/by/4.0/).

4.”In this study, we performed a single-cell transcriptome analysis of rice zygotes produced by an IVF system at six early developmental stages (15 min, 30 min, 1 h, 2 h, 4 h, and 6 h after gamete fusion)”. Are there any variations in nuclear fusion stage at the same time point?
5.P8. “genes/transcripts with different functional categories were upregulated in a sequential and stepwise manner during karyogamy in early zygotes”. This is for sure. But readers need to know the details about who comes first and who appears later in the time sequence.
6.P10. “’implying the change in the maternal:paternal transcript ratio was due to differences in parental alleles”. Does maternal transcripts degradation also contribute to the ratio changes?
7.P11. “paternal genome activation”. The concept is not widely accepted in the discussion of maternal-to zygotic transition. Zygotic genome activation or paternal allele transcription are usually used.
8.”changes to parental contributions”. parental contribution to what?
9.”maternal dominance of transcripts in the earliest developmental stage was converted into bi-parental mode from approximately 30 min’“1 h post- fertilization by de novo transcription from paternal allele during the progression of karyogamy in zygotes”. This conclusion seems not in line with previous report in rice. Adding brief discussions may be helpful.
10.”a tight relationship between a shift from maternal to bi-parental expression and karyogamy is crucial for progressing proper zygotic development and embryogenesis in plants”. I can’t find evidence for the conclusion in this work, better not to mention it although it is already concluded similarly in Arabidopsis work.
11.P17. Was 45 cycles used in all PCR experiments?

Reviewer 2

Advance summary and potential significance to field

Toda, et al. collect transcriptome data from single gametes for 6 h after gamete fusion in rice. There are two key contributions from this manuscript:
-evidence of fairly broad paternal transcript expression shortly after karyogamy (biological result)
-availability of transcriptome data during the initial events after gamete fusion (resource)

Overall, this work presents a remarkably early view at gene expression from the very beginning of plant development.

Comments for the author

Major concerns:
(1) The data is collected after artificial gamete fusion by electroporation.
Additional data and text would help to assess how well this in vitro technique mimics physiological development:

a) There were no controls to assess the effect of the electrofusion on gene expression. This makes it impossible to determine which of the expression changes are a response to electrofusion rather than karyogomy itself. RNA-seq data from a late timepoint (e.g. 6h) after mock gamete fusion would be an important and valuable control. The mock condition could be electrofusion without a sperm cell (only an egg), or better yet could be fusion with a sperm cell that has been inactivated in some way.

b) What is the current evidence to suggest that normal development is followed after in vitro gamete fusion? and are there any observations where the in vitro fused zygotes do not behave perfectly normally? For instance, does karyogamy after gamete fusion depend on similar genes as karyogamy in vivo? Does the
embryo continue to develop normally by morphology? And if so how far? (a complete plant? An early stage?) A more concrete and nuanced discussion of the in vitro technique would help the reader better interpret the findings.

(2) The data is heavy over-sequenced, which could artifically skew and inflate the differential expression analysis. The authors report ~50 million reads per single cell in this study, but a cell the size of rice sperm, eggs, and zygotes might be expected to have only 50-800 thousand total polyA

© 2025. Published by The Company of Biologists under the terms of the Creative Commons Attribution License (https://creativecommons.org/licenses/by/4.0/).

RNA molecules; typical transcript counts from other single-cell studies are in the range of 2,000-50,000 UMIs (mRNA molecules) detected per cell. Basically, the current sequencing dept is likely 100x and very possibly more than 1000x over-sequenced.

Unfortunately, the kit the authors use does not allow transcript counting through UMIs or similar. PCR duplicates also cannot be identified by mapping redundancy because there is a random shearing step after the initial PCR cycles (so a single initial RNA molecule could contribute reads mapping at many
different positions). One significant finding of the paper (Fig 6, which shows the timing of paternal transcript detection) is unlikely to be affected by this artifact. However, almost every other result (DEGs etc) might be affected and I recommend the following analyses to assess the impact of this over-sequencing:
a) downsample the reads randomly to 10 million, 1 million, 100K, and 50K, and plot the number of detected genes vs read number for the single cell samples.
Also pool every sample at the same stage and then downsample the reads similarly (10 million, 1 million, etc but with mixed reads from all stage samples). I would do this analysis with a few key samples: egg cell, sperm cell, 6 h zygote.
These plots will be helpful to assess how much the samples are oversequenced
b) downsample the reads randomly for both the control egg cell and 6 h zygote (including down to 20K reads) and then repeat the differential expression analysis. How do the number and specific DEGs change after downsampling to varying degrees? This will help assess how sensitive the differential expression
analysis is to sample depth.
c) repeat differential expression analysis using a package that does not assume any statistical distribution (edgeR, DESeq, etc in the SCC package all assume a negative binomial). For instance, a smoothed bootstrap test could be applied instead. Assess how this changes the resulting DEGs.

Minor points:

Fig 1 and S2. These scatterplots are helpful to assess the data, but it would be better to plot them as log-log plots (similar to Fig 2) so that a greater dynamic range can be assessed.

Fig 2B -- it is very difficult to see the GFP signal in the merged GFP-brightfield images. Recommend scaling the GFP images prior to merging so that the signal is brighter.

Fig S1 and S2. Correlation coefficients are reported after excluding outliers, but the number and process of outlier exclusion is not reported. It may be preferred to calculate correlation after the log transformation, as this will be less sensitive to outliers but more sensitive to reproducibility in the low-medium abundance range (where most of the genes are).

<u>Reviewer 3</u>

Advance summary and potential significance to field

In this manuscript, Toda et al. report an impressive study that investigated the transcriptional dynamics during karyogamy in rice zygotes. The authors performed single-cell transcriptome analysis of rice zygotes at 15-minutes, 30-minutes, 1 hour, 2 hours, 4 hours and 6 hours after gamete fusion. They detected de novo expression in rice zygotes as soon as 15-minutes after gamete fusion. GO-term analyses suggest that genes expressed de novo in zygotes at different timepoints are involved in distinct developmental processes. Egg and sperm from different strains were then fused, and a SNP analysis was performed on the resulting zygotes at six timepoints ranging from 15 minutes to 6 hours. Consistent with early paternal genome activation, the proportion of paternal transcripts detected increased between 30 minutes and 1 hour. Paternal transcripts only made up ~35% of total transcripts at 6 hours suggesting that zygotic genome was not fully transcriptionally active or that maternal transcripts were not fully cleared at the last time point that was sampled.

Overall, this appears very high-quality data from a time series of zygotes, which is an impressive technical achievement and produced a valuable dataset. Their report also gives new insights into

© 2025. Published by The Company of Biologists under the terms of the Creative Commons Attribution License (https://creativecommons.org/licenses/by/4.0/).

the dynamics of the maternal-to-zygotic transition in plants, which is interesting and well-described. In short, this is such a nice set of data and I think some more basic analyses and small changes to the text, would significantly improve the manuscript with relatively little effort. To help the authors improve their manuscript, I have listed major and minor points to be addressed below.

Comments for the author

Major points
-The authors identified more maternal transcripts in early stages compared to later stages. In my opinion, these should not be referred to as maternally expressed genes (MEGs) because this implies that they are expressed from the maternal allele in zygotes. In general, it is difficult to conclude whether maternally biased transcripts are due to maternal inheritance or maternal expression. Based on the data presented and the early time points sampled, it seems that most maternal transcript bias is due to carryover from the egg, which is intuitive. Accordingly, I think some language about maternal "expression" in the Abstract, Results and Discussion should be changed.
-Related to the point above, inheritance of maternal transcripts is somewhat visible in Fig. S9B and Fig. S9C. These patterns would likely be clearer if the heatmaps were also plotted as row-scaled instead of log10(TPM + 1). The authors may also consider showing these data as a main figure. These dynamics are quite interesting and, based on what I know, illustrate the most detailed view of the MZT in plants presented so far. It could also be interesting to run GO-term analyses on these various sets of "MEGs" (and PEGs) like what was done for the persistent upregulated and downregulated DEGs above.
-I also question the accuracy of the statement in the Discussion section suggesting that the timing of the MZT varies among plant species based on what was observed in the Zhao et al. 2019, Dev. Cell report. Zhao et al. analyzed early and late zygote stages, but the submitted manuscript focused on early zygotes (15 minutes to 6 hours after fusion; whereas it takes ~17-20 hours for the first division). It seems that late-staged zygotes would have close to 50% maternal/paternal transcripts which would be similar to what is observed in Arabidopsis.
-Related to the point above, the authors should also compare and contrast their results with what was reported by Anderson et al., 2017, Dev. Cell who also examined transcriptome dynamics in early rice zygotes. In particular, Anderson et al. found that ~97% of genes had maternally biased transcript levels 2.5 hours after fertilization. In the current manuscript, there appear to be only ~77% and ~79% maternally biased reads at 2 and 4 hours after fusion. I could not figure out how many genes they analyzed for their SNP analysis of intersubspecific fusions, but based on the scatterplots in Fig. 6F,G, it appears much less than what was observed by Anderson et al. Were these differences due to how the zygotes were sampled, sequenced or analyzed?
-The authors also found ~650 DEGs that were continuously upregulated relative to the egg cell starting at different timepoints and ending at the 6hr timepoint. Although this seems low (especially because an FDR < 0.05 was used as their cutoff), it may reflect initial waves of zygotic genome activation. This would be consistent with the largest amount of upregulation occurring between the last two timepoints sampled (4h and 6h). Similarly, relatively few persistent downregulated DEGs were identified. Does this mean that the sampled time points were too early to observe the degradation of most maternal transcripts? In my opinion, these points deserve more discussion in the text.

Minor points
-Based on correlations and bioanalyzer traces, the RNA-seq data looks very good. However, it would be useful to visualize the individual transcriptomes in a PCA plot to better visualize the variation in gene expression that takes place after karyogamy especially in relation to the gametes.
-I don't understand why the authors used semi-qPCR instead of RT-qPCR for their validations, but probably okay.
-How were the timepoints precisely determined? Did the authors watch H2B-GFP from sperm under the microscope after fusion and then select each individually? Or did they establish timepoints and then use these without watching directly? This was not clear to me in the Methods.
-I didn't understand the significance that persistent upregulated DEGs encoded proteins with more protein-protein interactions. The authors could discuss this in more detail or move from Fig. 4 to Supplemental Material.
-It was impossible to read the labels of PPI network in Fig. 4
-The authors should put the total number of genes analyzed for each analysis shown; for example, Add number of N.S. genes in Fig. 2D-I, total number of P1-P5 and S1-S6 transcripts in Fig. 3B,D and

© 2025. Published by The Company of Biologists under the terms of the Creative Commons Attribution License (https://creativecommons.org/licenses/by/4.0/).

Fig. 5B,E, and especially Fig. 6C-H. As mentioned above, I could not determine how many genes they tested for biased transcript levels.

First revision

Author response to reviewer's comments

We have provided additional results and substantially revised the manuscript to incorporate the editor's and reviewer's comments. Herein, we send the revised manuscript entitled "Transcriptional dynamics during karyogamy in rice zygotes" for your consideration of its suitability for publication in Development. Changes from the former version of the manuscript are presented as red-colored font.

Responses to reviewer's comments are presented below.

Responses to reviewer #1
We are much thankful for reviewer's comments and have revised the manuscript according to the reviewer's comments. Item number corresponds to the reviewer's comments for the former version of our manuscript. Changes from the former version of the manuscript are presented as red-colored font.

Major concerns:
1. [Reviewer comment] In this work authors discussed "de novo and altered gene expression in zygotes from 15 min postfertilization". According to Fig 2, at 15 min after gamete fusion the karyogamy is not yet initiated and thus zygote genome is not yet established. Generally, it is considered that the transcripts in zygote are either gamete-delivered during fertilization or de novo transcribed by zygote genome. If authors' conclusion was true, then, there should be the third part of the transcripts, that is paternal genome or maternal genome transcription after fertilization and before nuclear fusion and zygote genome establishment. This can be an important new finding or just a character of in vitro fertilization. Please carefully think about it and discuss this issue in the manuscript.
[Response] Thank you for the comment. As the reviewer indicated, we confirmed the upregulation of genes in early zygotes is induced by gamete fusion and karyogamy, not by stimuli from electrofusion and subsequent cell culture which are utilized in IVF procedures. We added Fig. S18 showing expression profiles of egg cells subjected to mock gamete fusion without sperm cells and incubation for 4 h. The result indicated that expression profiles of the transcripts in egg cells under mock condition were similar to those in egg cells, suggesting that the expression changes are not a response to electrofusion, but rather are derived from gamete fusion and karyogamy itself. We added the description in Discussion section (Page 12 Line 262 to Page 13 Line 269).

2. [Reviewer comment] To confirm de novo transcription, authors used semi-quantitative RT-PCR, but detailed conditions for perform this experiment are not well described. This is important for readers to understand how the conclusion was made. [Response] Thank you for the comment. Semi-quantitative RT-PCR was performed at two laboratories. Although the cDNA used in this experiment was the same, the experimental conditions were slightly modified due to differences in the experimental equipment. As the reviewer indicated, we added the detailed descriptions in Methods (Page 18 Line 418 to Page 19 Line 429).

3. [Reviewer comment] Authors mentioned that "paternal allelic expression was initiated from 1 h after gamete fusion" when male and female gamete nuclei underwent fusion. Normally, paternal or maternal allelic expression is used to describe gene expression after zygotic genome activation. However, at 1 h after gamete fusion zygotic genome is not yet established, not to mention zygotic genome activation. Therefore, this description may cause confusion.
[Response] Thank you for the comment. According to the reviewer's comments, we modified the text from "paternal allelic expression" to "transcription from paternal allele" in Page 11 Line 240.

© 2025. Published by The Company of Biologists under the terms of the Creative Commons Attribution License (https://creativecommons.org/licenses/by/4.0/).

4. [Reviewer comment] According to Fig 2, 15-30 min after gamete fusion is very important period to reveal what happens before karyogamy and after gamete fusion. Up to now, almost nothing has been known about the mechanism underlying gamete nuclear fusion except some mutants interrupted this process. Adding a section to analyses how many and what type of genes expressed or upregulated in this period would be helpful for further investigations.
[Response] Thank you for the suggestion. According to the reviewer's comments, we added Supplementary Tables which show expression profiles of up- and down-regulated DEGs during karyogamy in rice zygotes (Table S5 and S6), and these newly added Tables are cited in Page 7 Line 145.

Minor concerns:
1. [Reviewer comment] In Summary statement: "in plant zygotes" can be deleted since rice zygote is already mentioned or move "rice zygote" to the end of the sentence.
[Response] Thank you for the suggestion. According to the reviewer's comments, we modified the text in Summary statement (Page 2).

2. [Reviewer comment] P3, "implying the mixing of the male and female genomes before the first cell division". The word "mixing" is not properly used here. Maybe, integrating is better.
[Response] Thank you for the suggestion. According to the reviewer's comments, we modified the word in Page 3 Line 51 and Page 14 Line 301.

3. [Reviewer comment] P5, "Although karyogamy seems to be essential for proper embryogenesis". Recently, several reports revealed that parthenogenesis is possible without karyogamy.
[Response] Thank you for the comment. According to the reviewer's comments, we modified the text in Page 4 Line 93-94.

4. [Reviewer comment] "In this study, we performed a single-cell transcriptome analysis of rice zygotes produced by an IVF system at six early developmental stages (15 min, 30 min, 1 h, 2 h, 4 h, and 6 h after gamete fusion)". Are there any variations in nuclear fusion stage at the same time point?
[Response] Thank you for the comment. As the reviewer indicated, there are some variations in nuclear fusion stage at the same time point. We selected zygotes showing typical karyogamy progression (Fig. 2A-C; Ohnishi et al., 2014) and excluded zygotes showing late nuclear fusion. The added text is presented in Page 16 Line 356-360.

5. [Reviewer comment] P8. "genes/transcripts with different functional categories were upregulated in a sequential and stepwise manner during karyogamy in early zygotes". This is for sure. But readers need to know the details about who comes first and who appears later in the time sequence.
[Response] Thank you for the comment. According to the reviewer's comments, we added more detailed description about who comes first and who appears later in Page 8 Line 184 to Page 9 Line 186.

6. [Reviewer comment] P10. "…implying the change in the maternal:paternal transcript ratio was due to differences in parental alleles". Does maternal transcripts degradation also contribute to the ratio changes?
[Response] Thank you for the suggestion. As the reviewer indicated, transcriptional suppression of maternal allele and degradation of maternal transcripts may also affect the reduced proportion of maternal reads.
Therefore, we added the description in Page 10 Line 216-218.

7. [Reviewer comment] P11. "paternal genome activation". The concept is not widely accepted in the discussion of maternal-to zygotic transition.
Zygotic genome activation or paternal allele transcription are usually used.
[Response] Thank you for the comment. According to the reviewer's comments, we modified the text in Page 2 Line 33, Page 3 Line 58, and Page 12 Line 258.

8. [Reviewer comment] "changes to parental contributions". Parental contribution to what?
[Response] Thank you for the comment. According to the reviewer's comments, we modified the

© 2025. Published by The Company of Biologists under the terms of the Creative Commons Attribution License (https://creativecommons.org/licenses/by/4.0/).

text in Page 12 Line 247-250.

9. [Reviewer comment] “maternal dominance of transcripts in the earliest developmental stage was converted into bi-parental mode from approximately 30 min-1 h post-fertilization by de novo transcription from paternal allele during the progression of karyogamy in zygotes”. This conclusion seems not in line with previous report in rice. Adding brief discussions may be helpful.
[Response] Thank you for the comment. We added the description for the previous report in rice (Anderson et al., 2017), especially about maternal dominance of transcripts in early zygotes. The added text is presented in Page 13 Line 285 to Page 14 Line 296.

10. [Reviewer comment] “a tight relationship between a shift from maternal to bi-parental expression and karyogamy is crucial for progressing proper zygotic development and embryogenesis in plants”. I can’t find evidence for the conclusion in this work, better not to mention it although it is already concluded similarly in Arabidopsis work.
[Response] Thank you for the comment. We agree with the reviewer’s comment and the description was removed from the text.

11. [Reviewer comment] P17. Was 45 cycles used in all PCR experiments? [Response] Thank you for the comment. In RT-qPCR, 45 cycles were used in all experiments. On the other hand, 30 or 35 cycles were used for semi-quantitative RT-PCR. As for the PCR cycle, we modified the text in Page 18 Line 418 to Page 19 Line 429 and added explanation in figure legend of Fig. 3.

Other points:
We added the descriptions for suitability and validity of in vitro fertilization system (utilization of zygotes produced by IVF system) in Introduction section (Page 5 Line 97-103).

To assess TPM values in the low-medium abundance range where most of the transcripts are, scatter plots with log conversion represent in new Fig. S2, S6 and S15.
We conducted additional data analyses using downsampled reads to assess sequencing depth of RNA-seq data for the gametes and zygotes. The number of detectable transcripts and DEGs were counted and compared between pre-processed and downsampled reads (new Fig. S3 and S8), suggesting that the sequencing depth in this study is in appropriate range for analysis. The description for the results in Fig. S3 was added in Page 6 Line 131 to Page 7 Line 134, and Fig. S8 is cited in Page 7 Line 145.

To visualize the individual transcriptomes, we added PCA plots in new Fig. S4 and added the text in Page 7 Line 134-136.

We added heat maps for row-scaled allelic TPM values of 3632 MEGs and 66 PEGs (detected in Fig. 6C-H) in NK and KN zygotes at 15 min-6 h after gamete fusion as Fig. 6I-L. The texts for these results were added in Page 10 Line 229-235.

Responses to reviewer #2
We are much thankful for reviewer's comments and have revised the manuscript according to the reviewer’s comments. Item number corresponds to the reviewer’s comments for the former version of our manuscript. Changes from the former version of the manuscript are presented as red-colored font.

Major concerns:
1. [Reviewer comment] The data is collected after artificial gamete fusion by electroporation. Additional data and text would help to assess how well this in vitro technique mimics physiological development:
[Response] Thank you for the comment. Responses to reviewer’s comments (1a and 1b) are presented below.

a) [Reviewer comment] There were no controls to assess the effect of the electrofusion on gene expression. This makes it impossible to determine which of the expression changes are a response to electrofusion rather than karyogomy itself. RNA-seq data from a late timepoint (e.g. 6h) after mock gamete fusion would be an important and valuable control. The mock condition could be

© 2025. Published by The Company of Biologists under the terms of the Creative Commons Attribution License (https://creativecommons.org/licenses/by/4.0/).

electrofusion without a sperm cell (only an egg), or better yet could be fusion with a sperm cell that has been inactivated in some way.
[Response] Thank you for the suggestion. To assess this point, three egg cells were subjected to mock gamete fusion without sperm cells and sampled individually after incubation for 4 h. Since our previous study has identified fertilization-induced genes in rice zygotes (Abiko et al., 2013), we selected the representative transcripts in the fertilization-induced genes and confirmed the TPM values in the present study (Fig. S18A), showing their upregulations after gamete fusion. To compare their expression profiles among egg cells, egg cells under mock condition and zygotes at 4 h after gamete fusion, we performed semi-quantitative RT- PCR as an alternative of RNA-seq analysis. The result indicated that expression profiles of the transcripts in egg cells under mock condition were similar to those in egg cells (Fig. S18B), suggesting that the expression changes are not a response to electrofusion, but rather are derived from gamete fusion and karyogamy itself. We added the description in Discussion (Page 12 Line 262 to Page 13 Line 269). We would appreciate if the reviewer accepted our explanation and additional analysis.

b) [Reviewer comment] What is the current evidence to suggest that normal development is followed after in vitro gamete fusion? and are there any observations where the in vitro fused zygotes do not behave perfectly normally? For instance, does karyogamy after gamete fusion depend on similar genes as karyogamy in vivo? Does the embryo continue to develop normally by morphology? And if so how far? (a complete plant? An early stage?) A more concrete and nuanced discussion of the in vitro technique would help the reader better interpret the findings.
[Response] Thank you for the comment. According to the reviewer’s suggestion, we added the descriptions for suitability and validity of in vitro fertilization system (utilization of zygotes produced by IVF system) in Introduction section (Page 5 Line 97-103).

2. [Reviewer comment] The data is heavy over-sequenced, which could artifically skew and inflate the differential expression analysis. The authors report ~50 million reads per single cell in this study, but a cell the size of rice sperm, eggs, and zygotes might be expected to have only 50-800 thousand total polyA RNA molecules; typical transcript counts from other single-cell studies are in the range of 2,000-50,000 UMIs (mRNA molecules) detected per cell. Basically, the current sequencing dept is likely 100x and very possibly more than 1000x over-sequenced. Unfortunately, the kit the authors use does not allow transcript counting through UMIs or similar. PCR duplicates also cannot be identified by mapping redundancy because there is a random shearing step after the initial PCR cycles (so a single initial RNA molecule could contribute reads mapping at many different positions). One significant finding of the paper (Fig 6, which shows the timing of paternal transcript detection) is unlikely to be affected by this artifact. However, almost every other result (DEGs etc) might be affected and I recommend the following analyses to assess the impact of this over-sequencing:
[Response] Thank you for the comment. Responses to reviewer’s comments (2a-c) are presented below.

a) [Reviewer comment] downsample the reads randomly to 10 million, 1 million, 100K, and 50K, and plot the number of detected genes vs read number for the single cell samples. Also pool every sample at the same stage and then downsample the reads similarly (10 million, 1 million, etc but with mixed reads from all stage samples). I would do this analysis with a few key samples: egg cell, sperm cell, 6 h zygote. These plots will be helpful to assess how much the samples are oversequenced
[Response] Thank you for the comment. According to the reviewer’s comments, the pre-processed reads were randomly downsampled to 20 million, 10 million, 5 million, 2 million, 1 million, 500K, 200K, 100K, and 50K. Plots of the number of transcripts (TPM > 0) vs read number for single cell samples and mixed samples showed a sharp decrease in the number of detected transcripts less than 5 million reads. These data suggested that our sequencing data is not 100x or 1000x over-sequencing as pointed out by the reviewer. These data were presented as new Fig. S3 and the descriptions for these results were added in the text (Page 6 Line 131 to Page 7 Line 134).

b) [Reviewer comment] downsample the reads randomly for both the control egg cell and 6 h zygote (including down to 20K reads) and then repeat the differential expression analysis. How do the number and specific DEGs change after downsampling to varying degrees? This will help assess how sensitive the differential expression analysis is to sample depth.
[Response] Thank you for the comment. According to the reviewer’s comments, we performed the

© 2025. Published by The Company of Biologists under the terms of the Creative Commons Attribution License (https://creativecommons.org/licenses/by/4.0/).

differential expression analysis of the pre- processed and downsampled data (egg cell vs 6 h zygote) using TCC. The results showed that the number of DEGs and specific DEGs were largely stable over 5 million reads. These additional data were represented as new Fig. S8, and the newly added Figure is cited in Page 7 Line 145.

c) [Reviewer comment] repeat differential expression analysis using a package that does not assume any statistical distribution (edgeR, DESeq, etc in the SCC package all assume a negative binomial). For instance, a smoothed bootstrap test could be applied instead. Assess how this changes the resulting DEGs.
[Response] Thank you for the comment. According to the reviewer's comments, we performed a differential expression analysis using samr (v3.0; Li and Tibshirani, 2013) as non-parametric approach. The option nperm = 2000 was applied for stable detection of the resulting DEGs (relatively stable results were obtained even nperm = 1000). The resulting DEGs were confirmed by fertilization-induced transcripts in Fig. S18A. As a result, the transcripts that were clearly upregulated in early stage of zygotes (compared to egg cells) were detected as DEGs in both TCC and samr (FDR < 0.05), while those slightly upregulated in early zygotes were detected as DEGs only in TCC. We are considering that TCC seems to be slightly sensitive to detect DEGs in the RNA-seq data of this study, and application of multiple statistical analyses would be effective to detect transcripts with more clearly significant differences. As for this point, we added the description to Page 13 Line 269-273.

Minor concerns:
1. [Reviewer comment] Fig 1 and S2. These scatterplots are helpful to assess the data, but it would be better to plot them as log-log plots (similar to Fig 2) so that a greater dynamic range can be assessed.
[Response] Thank you for the suggestion. As the reviewer indicated, we added log-log plots in new Fig. S2, S6 and S15. One of the aims of these scatterplots is to calculate correlation efficient of TPM values for evaluating a reliability of RNA-seq data. Therefore, we keep these scatterplots without log conversion in Fig. 1, S5 and S14. We would appreciate if the reviewer accepted our explanation.

2. [Reviewer comment] Fig 2B -- it is very difficult to see the GFP signal in the merged GFP-brightfield images. Recommend scaling the GFP images prior to merging so that the signal is brighter.
[Response] Thank you for the comment. According to the reviewer's comments, we modified Fig. 2B to show the GFP signal more clearly in the merged GFP-brightfield images.

3. [Reviewer comment] Fig S1 and S2. Correlation coefficients are reported after excluding outliers, but the number and process of outlier exclusion is not reported. It may be preferred to calculate correlation after the log transformation, as this will be less sensitive to outliers but more sensitive to reproducibility in the low-medium abundance range (where most of the genes are).
[Response] Thank you for the suggestion. We described the numbers of the excluded transcripts as outliers and modified the text in figure legends of Fig. 1, S5 and S14.

Other points:
To visualize the individual transcriptomes, we added PCA plots in new Fig. S4 and added the text in Page 7 Line 134-136.
We added Supplementary Tables which show expression profiles of up- and down-regulated DEGs during karyogamy in rice zygotes (Table S5 and S6), and these newly added Tables are cited in Page 7 Line 145.

We added heat maps for row-scaled allelic TPM values of 3632 MEGs and 66 PEGs (detected in Fig. 6C-H) in NK and KN zygotes at 15 min-6 h after gamete fusion as Fig. 6I-L. The texts for these results were added in Page 10 Line 229-235.

We added the description for the previous report in rice (Anderson et al., 2017), especially about maternal dominance of transcripts in early zygotes. The text is presented in Page 13 Line 285 to Page 14 Line 296.

© 2025. Published by The Company of Biologists under the terms of the Creative Commons Attribution License (https://creativecommons.org/licenses/by/4.0/).

Responses to reviewer #3
We are much thankful for reviewer's comments and have revised the manuscript according to the reviewer's comments. Item number corresponds to the reviewer's comments for the former version of our manuscript. Changes from the former version of the manuscript are presented as red-colored font.

Major concerns:
1. [Reviewer comment] The authors identified more maternal transcripts in early stages compared to later stages. In my opinion, these should not be referred to as maternally expressed genes (MEGs) because this implies that they are expressed from the maternal allele in zygotes. In general, it is difficult to conclude whether maternally biased transcripts are due to maternal inheritance or maternal expression. Based on the data presented and the early time points sampled, it seems that most maternal transcript bias is due to carryover from the egg, which is intuitive. Accordingly, I think some language about maternal "expression" in the Abstract, Results and Discussion should be changed.
[Response] Thank you for the comment. According to the reviewer's comment, we changed the text from "maternal expression" to "maternal- biased transcripts" in Abstract (Page 2 Line 36), Results (Page 10 Line 222-226) and Discussion (Page 12 Line 252).

2. [Reviewer comment] Related to the point above, inheritance of maternal transcripts is somewhat visible in Fig. S9B and Fig. S9C. These patterns would likely be clearer if the heatmaps were also plotted as row- scaled instead of log10(TPM + 1). The authors may also consider showing these data as a main figure. These dynamics are quite interesting and, based on what I know, illustrate the most detailed view of the MZT in plants presented so far. It could also be interesting to run GO-term analyses on these various sets of "MEGs" (and PEGs) like what was done for the persistent upregulated and downregulated DEGs above.
[Response] Thank you for the suggestion. According to the reviewer's comments, we added heat maps for row-scaled allelic TPM values of 3632 MEGs and 66 PEGs (detected in Fig. 6C-H) in NK and KN zygotes at 15 min-6 h after gamete fusion as Fig. 6I-L. In addition, we performed KEGG analysis on these various sets of MEGs (instead of GO analysis), resulting 'Metabolic pathways' or 'Ribosome' were specifically and commonly detected in the M2-M5 (Fig. S16). The texts as for these results were added in Page 10 Line 229-235 and Page 11 Line 238-239.

3. [Reviewer comment] I also question the accuracy of the statement in the Discussion section suggesting that the timing of the MZT varies among plant species based on what was observed in the Zhao et al. 2019, Dev. Cell report. Zhao et al. analyzed early and late zygote stages, but the submitted manuscript focused on early zygotes (15 minutes to 6 hours after fusion; whereas it takes ~17-20 hours for the first division). It seems that late-staged zygotes would have close to 50% maternal/paternal transcripts which would be similar to what is observed in Arabidopsis.
[Response] Thank you for the comment. As the reviewer indicated, it seems that middle or late-staged zygotes would have close to 50% maternal/paternal transcripts in rice; however, we do not show any evidence in this study. What we focus on here is the differences in parental contribution to early stage of zygotes among plant species. In Arabidopsis, maternally biased transcripts occupied in early stage of zygotes and genome-wide transition from maternal-biased transcripts to bi-parental allele expression reportedly occurs between early and late stage of zygotes. On the other hands, our study indicates that maternal dominance of transcripts starts to convert into bi-parental mode during early stage of rice zygotes. Considered to next reviewer's comment 4, we removed this description to focus on the discussion regarding to Anderson et al. (2017) and our study. We would appreciate if the reviewer accepted our explanation.

4. [Reviewer comment] Related to the point above, the authors should also compare and contrast their results with what was reported by Anderson et al., 2017, Dev. Cell who also examined transcriptome dynamics in early rice zygotes. In particular, Anderson et al. found that ~97% of genes had maternally biased transcript levels 2.5 hours after fertilization. In the current manuscript, there appear to be only ~77% and ~79% maternally biased reads at 2 and 4 hours after fusion. I could not figure out how many genes they analyzed for their SNP analysis of intersubspecific fusions, but based on the scatterplots in Fig. 6F,G, it appears much less than what was observed by Anderson et al. Were these differences due to how the zygotes were sampled, sequenced or analyzed?
[Response] Thank you for the comment. We analyzed 17,112 comparable transcripts in Fig. 6. As

© 2025. Published by The Company of Biologists under the terms of the Creative Commons Attribution License (https://creativecommons.org/licenses/by/4.0/).

the reviewer indicated, parental contribution in rice zygotes has been previously investigated and maternal dominance of transcripts was detected in hybrid zygotes at 2.5 h after pollination (Anderson et al., 2017). Considering the duration from pollination to fertilization, it is assumed that the result corresponds to that in zygotes at 15 min-1 h after gamete fusion in this study (Fig. 6). Alternatively, putative low pollination efficiency may become a reason for partial contamination of egg cells during the isolation procedures of pollinated egg cells (zygotes), resulting in the increase of maternal reads. The added description is presented in Page 13 Line 285 to Page 14 Line 296.

5. [Reviewer comment] The authors also found ~650 DEGs that were continuously upregulated relative to the egg cell starting at different timepoints and ending at the 6hr timepoint. Although this seems low (especially because an FDR < 0.05 was used as their cutoff), it may reflect initial waves of zygotic genome activation. This would be consistent with the largest amount of upregulation occurring between the last two timepoints sampled (4h and 6h). Similarly, relatively few persistent downregulated DEGs were identified. Does this mean that the sampled time points were too early to observe the degradation of most maternal transcripts? In my opinion, these points deserve more discussion in the text.
[Response] Thank you for the comment. As the reviewer indicated, the number of both persistent up- and down-regulated DEGs between the last two points were relatively higher than the early timepoints. The main point of our analysis is to show that persistent or stage-specific up-/down-regulation occur even in the early stages of zygotes. On the other hands, as the reviewer pointed out, it is also important to investigate the transcriptional dynamics of zygotes at middle or late developmental stage. We added such future perspectives in Discussion section (Page 13 Line 296-300).

Minor concerns:
1. [Reviewer comment] Based on correlations and bioanalyzer traces, the RNA-seq data looks very good. However, it would be useful to visualize the individual transcriptomes in a PCA plot to better visualize the variation in gene expression that takes place after karyogamy especially in relation to the gametes.
[Response] Thank you for the suggestion. According to the reviewer's comments, we added PCA plots in new Fig. S4 and added the text in Page 7 Line 134-136.

2. [Reviewer comment] I don't understand why the authors used semi- qPCR instead of RT-qPCR for their validations, but probably okay.
[Response] Thank you for the comment. The main purpose of the qPCR analysis in Fig. 3 is to compare the expression levels of each transcript between egg cell and each stage of zygotes. Since most of the transcripts analyzed in Fig. 3 are rarely expressed in egg cells, we considered it would be better to use semi-qPCR instead of the $2^{-\Delta\Delta Ct}$ method RT- qPCR for validation. We would be grateful if the reviewer accepts our explanation.

3. [Reviewer comment] How were the timepoints precisely determined? Did the authors watch H2B-GFP from sperm under the microscope after fusion and then select each individually? Or did they establish timepoints and then use these without watching directly? This was not clear to me in the Methods.
[Response] Thank you for the comment. As the reviewer indicated, we observed H2B-GFP signals from sperm cells under a microscope during incubation of the zygotes and then collect each individually. We added this explanation in Methods (Page 16 Line 356-360).

4. [Reviewer comment] I didn't understand the significance that persistent upregulated DEGs encoded proteins with more protein-protein interactions. The authors could discuss this in more detail or move from Fig. 4 to Supplemental Material.
[Response] Thank you for the comment. According to the reviewer's comments, we moved it to Supplementary information (Fig. S11).

5. [Reviewer comment] It was impossible to read the labels of PPI network in Fig. 4
[Response] Thank you for the comment. After moving it to Supplementary information (Fig. S11), we displayed this PPI network as large as possible. In addition, we indicated the KEGG pathway information in the diagram.

© 2025. Published by The Company of Biologists under the terms of the Creative Commons Attribution License (https://creativecommons.org/licenses/by/4.0/).

6. [Reviewer comment] The authors should put the total number of genes analyzed for each analysis shown; for example, Add number of N.S. genes in Fig. 2D-I, total number of P1-P5 and S1-S6 transcripts in Fig. 3B,D and Fig. 5B,E, and especially Fig. 6C-H. As mentioned above, I could not determine how many genes they tested for biased transcript levels.
[Response] Thank you for the comment. According to the reviewer's comments, we added the number of N.S. transcripts in Fig. 2D-I and Fig. 6C-H, and total number of categorized transcripts in Fig. 3B, D and Fig. 5B, D.

Other points:
We added the descriptions for suitability and validity of in vitro fertilization system (utilization of zygotes produced by IVF system) in Introduction section (Page 5 Line 97-103).

To assess TPM values in the low-medium abundance range where most of the transcripts are, scatter plots with log conversion represent in new Fig. S2, S6 and S15.

We conducted additional data analyses using downsampled reads to assess sequencing depth of RNA-seq data for the gametes and zygotes. The number of detectable transcripts and DEGs were counted and compared between pre-processed and downsampled reads (new Fig. S3 and S8), suggesting that the sequencing depth in this study is in appropriate range for analysis. The description for the results in Fig. S3 was added in Page 6 Line 131 to Page 7 Line 134, and Fig. S8 is cited in Page 7 Line 145.

We added Supplementary Tables which show expression profiles of up- and down-regulated DEGs during karyogamy in rice zygotes (Table S5 and S6), and these newly added Tables are cited in Page 7 Line 145.

We confirmed the upregulation of genes in early zygotes is induced by gamete fusion and karyogamy, not by stimuli from electrofusion and subsequent cell culture which are utilized in IVF procedures (new Fig. S18). The description was added in Discussion section (Page 12 Line 262 to Page 13 Line 269).

Second decision letter

MS ID#: DEVELOP/2024/203055

MS TITLE: Transcriptional dynamics during karyogamy in rice zygotes

AUTHORS: Erika Toda, Shizuka Koshimizu, Atsuko Kinoshita, Tetsuya Higashiyama, Takeshi Izawa, Kentaro Yano, Takashi Okamoto

I have now received all the referees reports on the above manuscript, and have reached a decision. The referees' comments are appended below, or you can access them online: please go to BenchPress and click on the 'Manuscripts with Decisions' queue in the Author Area.

The overall evaluation is positive and we would like to publish a revised manuscript in Development, provided that the referees' comments can be satisfactorily addressed. You will see that one reviewer has serious concerns about the oversequencing potential. This reviewer provides a reasonable way to deal with this concern and we would expect that you attempt the data analyses they suggest. If your results do change with the analysis, it will be important to use the more accurate sequence analysis pipeline. Please attend to all of the reviewers' comments in your revised manuscript and detail them in your point-by-point response. If you do not agree with any of their criticisms or suggestions explain clearly why this is so. If it would be helpful, you are welcome to contact us to discuss your revision in greater detail. Please send us a point-by-point response indicating your plans for addressing the referees' comments, and we will look over this and provide further guidance.

© 2025. Published by The Company of Biologists under the terms of the Creative Commons Attribution License (https://creativecommons.org/licenses/by/4.0/).

Reviewer 2

Advance summary and potential significance to field

Repeated from last review, as the significance has not changed in this draft:
Toda, et al. collect transcriptome data from single gametes for 6 h after gamete fusion in rice. There are two key contributions from this manuscript:
-evidence of fairly broad paternal transcript expression shortly after karyogamy (biological result)
-availability of transcriptome data during the initial events after gamete fusion (resource)

Overall, this work presents a remarkably early view at gene expression from the very beginning of plant development.

Comments for the author

The authors have added analyses and data that have helped address my concerns.
However, one major statistical concern still remains that I think is import to resolve prior to publication. The additions of Fig S2, S3, and S8 make it much easier to assess the amount of oversequencing. While 50-80 million reads were obtained per cell (Fig. S3A), there is not a major drop in the number of genes detected until the reads were downsampled to ~1 million. There is also no changed in detected DEGs until below 1 million transcripts were subsampled (Fig. S8). When data is thrown out and no information is lost, it is a clear sign of oversequencing. I think my original estimate of >100x oversequencing is consistent with these plots (certain at least 10x). Major assumptions of DESeq2 are violated in this situation, and it is not statistically appropriate for these oversequenced data.

The authors re-ran their analysis using samr, which would be less prone to artifacts from oversequencing; however, they do not report the results of this analysis in any meaningful way. How much did samr reduce the number of detected DEGs? how does the use of samr change the results? If there is a big discrepancy (I would expect far fewer genes from samr given the evidence of oversequences in the new supplemental figures), samr is more appropriate and reliable and it calls into questions the initial analysis.

I would recommend the following:
-Report the number of DEGs when using samr -If the number of DEGs is only slightly reduced (e.g. 2 fold or so), then report this. In such case, it would be helpful to include the samr FDRs/p-values alongside the DESeq2 FDRs/p-values in the supplement -If the number of DEGs is drastically reduced, it suggests many of the "significant" genes in the current draft are an artifact of inappropriate statistics. I would recommend replacing DESeq2 entirely with samr or another non-parametric test and revising the results/text as needed. Any results that don't hold up are suspect.

Minor comments:
-Fig S3 -- should the legend in Fig S3 read "Number of expressed genes" rather than "transcripts"?

-"IVF system" (line 97) - define the acronym on first use (e.g. "In vitro fertilization (IVF)")

-Some of the response to reviewers was all put in the discussion, even when it presented new results and figures (e.g. the paragraph starting line 261). This text may be more appropriate in the results section. There was no issues interpreting the manuscript, so this is a minor point

Reviewer 3

Advance summary and potential significance to field

The authors have addressed my points, which together with addressing the concerns of other reviewers, improved their manuscript. Again, this manuscript describes an impressive study about transcriptional dynamics during karyogamy in rice zygotes, which gives new insights into the dynamics of the maternal-to-zygotic transition in plants. The additional analyses further demonstrate that this is high-quality data and will be a valuable dataset for the community.

© 2025. Published by The Company of Biologists under the terms of the Creative Commons Attribution License (https://creativecommons.org/licenses/by/4.0/).

Comments for the author

In my opinion, no further revisions are necessary.

Second revision

Author response to reviewer's comments

We have provided additional results and revised the manuscript to incorporate the editor's and reviewer's comments. Herein, we send the revised manuscript entitled "Transcriptional dynamics during karyogamy in rice zygotes" for your consideration of its suitability for publication in Development. Changes from the former version of the manuscript are presented as red-colored font.

Responses to reviewer's comments are presented below.

Responses to reviewer #2

We are much thankful for reviewer's comments and have revised the manuscript according to the reviewer's comments. Item number corresponds to the reviewer's comments for the former version of our manuscript. Changes from the former version of the manuscript are presented as red-colored font.

Major concerns:

1. [Reviewer comment] Report the number of DEGs when using samr -If the number of DEGs is only slightly reduced (e.g. 2 fold or so), then report this. In such case, it would be helpful to include the samr FDRs/p-values alongside the DESeq2 FDRs/p-values in the supplement -If the number of DEGs is drastically reduced, it suggests many of the "significant" genes in the current draft are an artifact of inappropriate statistics. I would recommend replacing DESeq2 entirely with samr or another non-parametric test and revising the results/text as needed. Any results that don't hold up are suspect.

[Response] Thank you for the comment. According to the reviewer's comments, we calculated the number of DEGs using samr. The result showed that the number of DEGs calculated by samr (FDR < 0.05) was not much different from that calculated by TCC (edgeR; FDR < 0.05). We believe that this result addresses the reviewer's statistical concerns. The additional data was presented as Fig. S18C, and the newly added figure is cited in Page 13 Line 271.

Minor concerns:

1. [Reviewer comment] Fig S3 -- should the legend in Fig S3 read "Number of expressed genes" rather than "transcripts"?

[Response] Thank you for the comment. According to the reviewer's comments, we modified the legend in Fig. S3 from "Number of detectable transcripts" to "Number of expressed genes".

2. [Reviewer comment] "IVF system" (line 97) - define the acronym on first use (e.g. "In vitro fertilization (IVF)")

[Response] Thank you for the comment. We have defined the acronym on first use in Page 3 Line 68.

3. [Reviewer comment] Some of the response to reviewers was all put in the discussion, even when

© 2025. Published by The Company of Biologists under the terms of the Creative Commons Attribution License (https://creativecommons.org/licenses/by/4.0/).

it presented new results and figures (e.g. the paragraph starting line 261). This text may be more appropriate in the results section. There was no issues interpreting the manuscript, so this is a minor point

[Response] Thank you for the comment. As the reviewer suggested, the text associated with the results may generally be more appropriate in the Results section. On the other hand, we are considering that this paragraph (starting line 262) explains the appropriateness of the methods for identifying DEGs, and it would provide additional information to address concerns that some readers may have. We have therefore included the text in the Discussion section. We would be grateful if the reviewer accepted these explanations.

Third decision letter

MS ID#: DEVELOP/2024/204497R1

MS TITLE: Transcriptional dynamics during karyogamy in rice zygotes

AUTHORS: Erika Toda, Shizuka Koshimizu, Atsuko Kinoshita, Tetsuya Higashiyama, Takeshi Izawa, Kentaro Yano, Takashi Okamoto
ARTICLE TYPE: Research Article

I am happy to tell you that your manuscript has been accepted for publication in Development, pending our standard publication integrity checks.

© 2025. Published by The Company of Biologists under the terms of the Creative Commons Attribution License (https://creativecommons.org/licenses/by/4.0/).
